# Microglial Deubiquitinase OTUD7B Stabilizes STAT3 to Promote Neuroinflammation and Cognitive Decline in Alzheimer's Disease

**DOI:** 10.1002/advs.202523043

**Published:** 2026-08-19

**Authors:** Luyao Li, Hao Tang, Qin Yu, Yuyang Zhang, Binglu Shi, Ying Kong, Leiyu Xu, Jingjing Shao, Chenghong Hu, Lingyu She, Zhe Wang, Haiyi Chen, Aleksandr V. Samorodov, Gang Cao, Xia Zhao, Yi Wang

**Affiliations:** ^1^ Zhejiang Provincial Key Laboratory of Anti‐Cancer Chinese Medicines and Natural Medicines School of Pharmacy Hangzhou Normal University Hangzhou Zhejiang China; ^2^ School of Pharmacy Zhejiang Chinese Medical University Hangzhou Zhejiang China; ^3^ College of Life and Environmental Sciences Hangzhou Normal University Hangzhou Zhejiang China; ^4^ School of Pharmacy Hangzhou Medical College Hangzhou Zhejiang China; ^5^ Department of Pharmacology Bashkir State Medical University Ufa Russia

**Keywords:** alzheimer's disease, deubiquitinating enzymes, microglial, neuroinflammation, OTUD7B, STAT3

## Abstract

Neuroinflammation driven by microglial activation is a defining feature of Alzheimer's disease (AD), yet the molecular mechanisms sustaining this proinflammatory state remain unclear. Here, we identify the deubiquitinase OTUD7B as a critical regulator of microglial activation and AD pathology. OTUD7B expression was markedly elevated in microglia from AD mouse models and human patient datasets. Genetic ablation of OTUD7B markedly attenuated microglial activation and cytokine release, alleviated neuronal injury, and improved cognitive performance in AD mice. Mechanistically, OTUD7B directly interacted with STAT3 and removed K48‐linked ubiquitin chains at lysine 283, thereby stabilizing STAT3, promoting its nuclear translocation, and enhancing transcription of proinflammatory mediators. Integrative transcriptomic analysis revealed that OTUD7B deficiency suppressed proinflammatory transcriptional programs in microglia. Together, these findings uncover an OTUD7B‐STAT3 signaling axis that sustains microglial‐driven neuroinflammation and identify OTUD7B as a potential therapeutic target for mitigating neurodegenerative pathology in AD.

## Graphical abstract

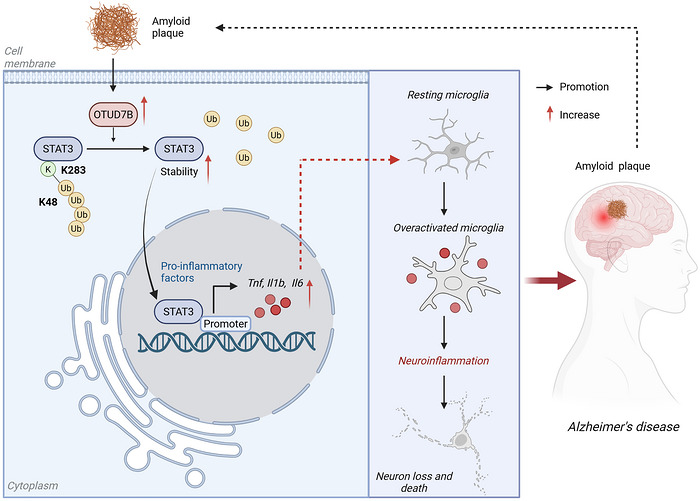

**Abbreviations**
AAV: adeno‐associated virus
AD: Alzheimer's disease
BSA: bovine serum albumin
ChIP: chromatin immunoprecipitation
CHX: cycloheximide
Co‐IP: Co‐immunoprecipitation
CQ: chloroquine
Cx3cr1: CX3C chemokine receptor 1
DUBs: deubiquitinating enzymes
ECL: enhanced chemiluminescence
ELISA: enzyme‐linked immunosorbent assay
GEO: gene expression datasets
GWAS: genome‐wide association studies
JAMMs: JAMM/MPN domain‐associated metallopeptidases
LC‐MS/MS: liquid chromatography‐tandem mass spectrometry
MJDs: Machado‐Joseph disease proteases
MWM: Morris Water Maze
NOR: Novel Object Recognition
OD: optical density
OTUD7B: ovarian tumor deubiquitinating enzyme 7B
OUT: ovarian tumor proteases
PBS: phosphate‐buffered saline
PFA: paraformaldehyde
RIPA: radio immunoprecipitation assay
SEM: standard error of the mean
SH2: Src Homology 2
STAT3: signal transducer and activator of transcription 3
UCHs: ubiquitin carboxyl‐terminal hydrolases
USPs: ubiquitin‐specific proteases
ZUP1s: Zinc finger‐containing ubiquitin peptidases

## 1 Introduction

Alzheimer's disease (AD) is the most prevalent form of dementia worldwide and represents one of the fastest‐growing public health challenges. With global population aging, AD‐related dementia cases in Europe are projected to double by 2050, while worldwide cases are expected to triple [1]. Despite decades of substantial research investment, the complex and multi‐layered pathophysiology of AD remains largely unresolved. A central pathological hallmark of AD is the accumulation of abnormal proteins within the central nervous system. Drug development guided by the amyloid cascade hypothesis has predominated; however, clinical trial failure rates remain as high as 98% [2, 3]. Effective treatments capable of mitigating or halting disease progression are still lacking, and amyloid‐targeting strategies demonstrate limited efficacy in symptomatic patients [4]. Consequently, investigating pathological mechanisms beyond Aβ deposition and identifying novel therapeutic targets have become urgent priorities in AD research [5].

In recent years, neuroinflammation has emerged as a key component of AD pathogenesis. This process constitutes a complex immune response to pathological stimuli in the brain, primarily involving microglia and astrocytes, whose activation can precede neurodegeneration and clinical symptoms [6]. Genome‐wide association studies (GWAS) have identified over 25 AD risk genes, many of which are linked to neuroinflammation or are specifically expressed in microglia [7]. Polymorphisms in these genes often affect microglial function, positioning microglia at the center of AD pathology [8]. Moreover, numerous risk loci encode proteins directly involved in glial function, reinforcing the critical role of neuroinflammation in disease progression [9]. Microglia, as resident macrophages of the central nervous system, not only constitute a vital component of innate immunity but also play essential roles in development, homeostasis, and various neuroinflammatory, neurodegenerative, neuropsychiatric, and neoplastic conditions [10, 11]. Therefore, modulating microglial immune responses represents a promising therapeutic strategy for AD [4].

Ubiquitination and its reverse process, deubiquitination, are key post‐translational protein modification systems in eukaryotic cells, regulating processes such as membrane trafficking, DNA repair, signal transduction, and protein degradation [12, 13]. Deubiquitinating enzymes (DUBs) maintain protein homeostasis by removing ubiquitin from substrates, ensuring precise reversibility of ubiquitination. Although the human genome encodes approximately 600 E3 ligases, it contains only about 100 DUBs, suggesting broader substrate specificity [14]. Increasing evidence indicates that dysregulation of the ubiquitination/deubiquitination network contributes to the pathogenesis of neurodegenerative diseases, highlighting DUBs as important molecular targets. Since the ubiquitin‐proteasome system and the autophagy‐lysosomal pathway are central to the clearance of misfolded or aggregated proteins, DUBs play critical regulatory roles in these processes. The DUB family is divided into seven subclasses, with the ovarian tumor proteases (OTU) family being the second largest. Dysfunction of OTU family members is associated with cancer, immune disorders, inflammation, and neurological diseases [15, 16].

Recent studies have highlighted the role of OTU family members in neurodegeneration. For example, OTUB1 colocalizes with α‐synuclein in Parkinson's disease models to form toxic aggregates and promotes AD progression by stabilizing Tau protein [17], whereas OTUD3 regulates Parkinson's disease‐associated neuroinflammation via cGAS ubiquitination [18]. A20 enhances cerebral ischemic tolerance by deubiquitinating RIP3 and modulates AD progression through NF‐κB signaling [19]. OTUD7B (also known as Cezanne), which shares a conserved OTU catalytic domain and structural similarity with OTUB1 and OTUD3, also acts as a critical regulator of inflammation by modulating multiple signaling cascades, including the mTORC2 and NF‐κB pathways [20, 21]. OTUD7B has been reported to participate in cell cycle regulation, tumorigenesis, and inflammatory responses by acting on various substrates, particularly in diseases involving the non‐canonical NF‐κB pathway [22]. However, its role in neurodegenerative diseases, especially in AD, has not been systematically investigated.

In this study, analysis of publicly available gene expression datasets (GEO) combined with polymerase chain reaction validation revealed significant upregulation of OTUD7B in AD microglia, suggesting its involvement in disease development. Functional studies demonstrated that OTUD7B deficiency attenuated microglial overactivation and inflammatory responses. Mechanistically, OTUD7B directly targeted the transcription factor, STAT3 (Signal transducer and activator of transcription 3), removing K48‐linked ubiquitin chains at K283, thereby stabilizing STAT3 and promoting transcription of downstream inflammatory factors, ultimately regulating microglial function. Collectively, these findings reveal, for the first time, a pivotal role for OTUD7B in AD‐associated neuroinflammation and suggest its potential as a therapeutic target.

## 2 Methods

### 2.1 Reagents

Aβ42(NH2‐DAEFRHDSGYEVHHQKLVFFAEDVGSNKGAIIGLMVGGVVIA‐COOH) was purchased from Ontores Biotechnologies (Hangzhou, China). Antibodies against STAT3(Cat#: 12640), p‐STAT3(Cat#: 9145), TNF‐α (Cat#: 370), IL‐1β (Cat#: 1224), GFAP (Cat#: 367), NeuN (Cat#: 9440), Iba1 (Cat#: 1719), BCL‐2 (Cat#: 1507), His‐tag (Cat#: 2365), Flag‐tag (Cat#: 14793), NF‐κB p65 (Cat#: 824), p‐NF‐κB p65 (Cat#: 3033), AlexaFluor594 (Cat#: 8889), and AlexaFluor488 (Cat#: 4412) were purchased from Cell Signaling Technology (Danvers, MA). Antibodies against OTUD7B (Cat#: 66276‐1‐Ig), KPNA3 (Cat#: 28050‐1‐AP), Myc‐tag (Cat#: 60003‐2‐Ig), GAPDH (Cat#: 60004‐1‐Ig), Lamin B (Cat#: 66095‐1‐Ig), and BAX (Cat#: 50599‐2‐Ig) were obtained from Proteintech (Wuhan, China). Annexin V‐FITC Apoptosis Detection Kit (Cat#: C1062), MTT (Cat#: ST316), anti‐rabbit IgG HRP (Cat#: A0208), anti‐mouse IgG HRP (Cat#: A0216), and BeyoMag Mouse IgG Magnetic Beads (Cat#: P2171) were ordered from Beyotime Biotechnology (Shanghai, China).

### 2.2 Animals and Treatment

Male C57BL/6J mice, weighing from 18–22 g, were from Gempharmatech Company (Nanjing, China). Microglia‐specific OTUD7B knockout mice (OTUD7B^CKO^) were generated by crossing OTUD7B^f/f^ mice with Cx3cr1(CX3C chemokine receptor 1)‐Cre mice, which were obtained from Gempharmatech Company. Animals were maintained in a pathogen‐free facility under controlled conditions (22°C ± 2°C, 50%–60% relative humidity, 12 h light/dark cycle) and provided with standard rodent chow supplied by the animal research center of Hangzhou Medical College.

This study employed two AD models: an acute pathological model generated by Aβ42 infusion in microglia‐specific OTUD7B knockout mice, and a chronic pathological model established via adeno‐associated virus (AAV) injection in 3×Tg mice carrying APP Swedish, MAPT P301L, and PSEN1 M146V mutations. All procedures were conducted in accordance with National Institutes of Health guidelines for the care and use of laboratory animals, ensuring humane treatment. All the animal experimental protocols were approved by the Animal Ethics Committee of Hangzhou Medical College (approval number: 2023–052).

#### 2.2.1 AAV‐Mediated Microglia‐Specific OTUD7B Knockout in 3×Tg Mice

To examine the role of OTUD7B in AD microglia, adeno‐associated virus (AAV9) vectors were constructed by BrainVTA (Wuhan, China), and the detailed information is shown in Table S1: a control vector (PT‐3048) and an shOTUD7B vector (PT‐11867). Each of these vectors was mixed with a microglia‐specific Cre‐expressing AAV (PT‐0606) at a 1:1 ratio and injected into the hippocampus of 8‐month‐old 3×Tg AD mice, thereby generating a model with microglia‐specific OTUD7B knockout (AAV‐shOTUD7B). Behavioral assessments were performed for four weeks post‐injection. Specifically, mice were anesthetized via intraperitoneal injection of 1% pentobarbital (40 mg/kg), and the cranial fur was shaved. Animals were secured in a stereotaxic apparatus, and a midline scalp incision was made to expose the anterior and posterior fontanelles. The microinjection syringe was positioned at the bregma, and coordinates were set to zero. Hippocampal CA1 injection coordinates were determined based on the Paxinos and Franklin atlas (anterior‐posterior: −2.0 mm; lateral: ±1.5 mm; depth from dura: 1.5 mm). Following the injection, the needle was retained for an additional 5 min before slow withdrawal.

#### 2.2.2 OTUD7B Knockout Mouse Model Infused With Aβ42

OTUD7B^f/f^ and OTUD7B^CKO^ mice (8 weeks old, 22–24 g) were randomly assigned to four groups (n = 10 per group): OTUD7B^f/f^ + Sham, OTUD7B^CKO^ + Sham, OTUD7B^f/f^ + Aβ (Aβ42‐infused OTUD7B^f/f^ mice), and OTUD7B^CKO^ + Aβ (Aβ42‐infused OTUD7B^CKO^ mice). For stereotaxic hippocampal injections, 1 µL of aggregated Aβ42 (5 µg/µL, incubated at 37°C for 7 days) was injected bilaterally into the CA1 region (0.5 µL per side). Sham‐operated OTUD7B^f/f^ mice received equivalent volumes of vehicle control (DMSO). Hippocampal CA1 injection coordinates were determined based on the Paxinos and Franklin atlas (anterior‐posterior: −2.0 mm; lateral: ±1.5 mm; depth from dura: 1.5 mm). Following the injection, the needle was retained for an additional 5 min before slow withdrawal [23, 24].

### 2.3 Behavior Test

Cognitive function in the experimental groups was assessed using the Morris Water Maze (MWM) and Novel Object Recognition (NOR) tests, following the established protocol [23]. Behavioral data were recorded and quantitatively analyzed using an automated tracking system (VisuTrack, Shanghai, China). For the MWM, mice were placed in a pool containing a hidden platform, and the latency to locate the platform was recorded (maximum trial duration: 60 s). Subsequently, the platform was removed for a 60‐second probe trial to assess spatial memory. During the probe trial, the number of crossings over the original platform location and the time spent in the target quadrant were recorded. For the NOR test, mice were initially exposed to two identical objects (A) in the testing apparatus for 5 min, and interactions with the objects were recorded. After 24 h, one familiar object was replaced with a novel object (B), and exploration was recorded for an additional 5 min. The Recognition Index (RI) was calculated as: RI (%) = New object/(New object + Old object) × 100.

### 2.4 Cell Culture and Transfection

The BV2 (Cat#: SNL‐155) and SH‐SY5Y (Cat#: SNL‐092) cells were obtained from Wuhan Shang'en Biotechnology Co., Ltd. (Wuhan, China), and the HEK‐293T cells (Cat#: GNHu17) were acquired from the National Center for Cell Culture (Shanghai, China). All the cell lines were maintained in DMEM (Cat#: 11995065, Gibco, Eggenstein, Germany) supplemented with 10% fetal bovine serum (FBS) and 1% antibiotics (streptomycin 100 µg/mL and penicillin 100 U/mL) (Cat#: 15140122, Gibco) at 37°C in a humidified incubator with 5% CO_2_. For stimulation experiments, Aβ was dissolved in DMSO to prepare a stock solution, which was diluted to the indicated concentrations in culture medium immediately before use. BV2 cells were treated with micromolar concentrations of Aβ to induce microglial activation, as reported in previous studies [25, 26].

siRNA sequences targeting mouse OTUD7B and STAT3 were designed by Genepharma (Shanghai, China) for gene silencing in BV2 cells (listed in Table S2). A scrambled siRNA served as the negative control. siRNA transfection was performed using Lipofectamine 2000 (Cat#: 168019, Thermo Fisher Scientific), and plasmid transfection was conducted with Lipofectamine 8000 (Cat#: C0533FT, Beyotime Biotechnology), according to the manufacturers’ protocols.

### 2.5 Western Blotting Analysis

Cells or tissues were lysed on ice in RIPA (Radioimmunoprecipitation Assay) buffer (Cat#: P0013B, Beyotime Biotechnology) supplemented with protease and phosphatase inhibitors. Lysates were centrifuged, and the supernatant was collected for protein denaturation. Proteins were separated by SDS‐PAGE and transferred to PVDF membranes at 300 V. Membranes were blocked with 5% skim milk at room temperature for 2 h, then incubated with specific primary antibodies at 4°C overnight. HRP‐conjugated secondary antibodies and enhanced chemiluminescence (ECL) reagent (Cat#: SQ201, Yeasen, Shanghai, China) were used to visualize immunoreactive bands. Band optical densities were quantified using ImageJ software (v1.54 g) and normalized to internal controls, including GAPDH (cytoplasmic protein), Lamin B (nuclear marker), and total protein levels for phosphorylated proteins.

### 2.6 Real‐Time Quantitative PCR

Total RNA was extracted from cultured BV2 cells or brain tissues using TRIzol reagent (Cat#: 15596026, Thermo Fisher Scientific) according to the manufacturer's instructions. RNA concentrations were determined with a spectrophotometer. cDNA was synthesized using the cDNA Synthesis Kit (Cat#: 11141ES60, Yeasen). Quantitative reverse transcription PCR (RT‐qPCR) was performed on a CFX96 Real‐Time PCR System (Bio‐Rad, Hercules, CA) using SYBR Green qPCR Master Mix II (Cat#: 731360ES50, Yeasen). Relative mRNA transcription levels were calculated using the 2^−ΔΔCt^ method, with *Gapdh* as the internal control. Primer sequences are provided in Table S3.

### 2.7 Flow Cytometry

Apoptosis was assessed by flow cytometry using Annexin V‐FITC and propidium iodide (PI) staining. Cells were stained with Annexin V‐FITC and PI according to the manufacturer's instructions (Cat#: C1062, Beyotime Biotechnology) and analyzed on a flow cytometer. The lower left quadrant represents viable cells, the lower right quadrant represents early apoptotic cells, the upper right quadrant represents late apoptotic cells, and the upper left quadrant represents necrotic cells. Apoptotic cells were quantified as the total apoptotic population, defined as the sum of early apoptotic cells and late apoptotic cells. Data are presented as the percentage of total apoptotic cells relative to the total cell population.

### 2.8 Chromatin Immunoprecipitation‐Quantitative PCR

Chromatin immunoprecipitation (ChIP) was carried out with the SimpleChIP Enzymatic Chromatin IP Kit (9003, Cell Signaling Technology, Danvers, MA) combined with magnetic beads. Quantitative PCR was subsequently performed using SYBR Green qPCR Master Mix II (Cat#: 731360ES50, Yeasen), with the human RPL30 gene serving as a positive control. Primers targeting additional promoter regions were designed using Oligo 7 software (oligo.net), and their sequences are listed in Table S4.

### 2.9 Immunocytochemistry (ICC)

BV2 cells were fixed with 4% paraformaldehyde (PFA) (Cat#: G1101, Servicebio, Wuhan, China) for 20 min, rinsed with phosphate‐buffered saline (PBS), and permeabilized with 0.1% Triton X‐100 (Cat#: GC204003, Servicebio) for 10 min. To block non‐specific antibody binding, cells were incubated in PBS containing 5% bovine serum albumin (BSA) for 1 h. Cells were incubated with primary antibodies at 4°C overnight, then washed with PBS and incubated with fluorescently labeled secondary antibodies at room temperature for 2 h. Nuclei were counterstained with DAPI (Cat#: P0131, Beyotime Biotechnology). Images were acquired using a Nikon A1 confocal microscope (Nikon, Tokyo, Japan).

### 2.10 Immunofluorescent Staining (IF)

Brain tissues were fixed in 4% PFA for 24 h, then incubated in 20% sucrose to remove residual PFA, followed by 30% sucrose until the tissues sank. Tissues were then embedded in OCT, and 15 µm sections were cut using a cryostat (Leica CM3050, Wetzlar, Germany). Sections were dried at room temperature for 1 h and washed three times with 1× PBS. For immunostaining, sections were permeabilized with 0.5% Triton X‐100 for 20 min and blocked with PBS containing 10% BSA for 1 h. Sections were incubated with primary antibodies at 4°C overnight, followed by incubation with fluorescently labeled secondary antibodies for 1 h at room temperature and counterstaining of nuclei with DAPI (Cat#: P0131, Beyotime Biotechnology). Images were acquired using a Nikon A1 confocal microscope (Nikon).

### 2.11 Liquid Chromatography‐Tandem Mass Spectrometry (LC‐MS/MS)

To identify potential OTUD7B binding targets, an LC‐MS/MS assay was employed. Briefly, OTUD7B was expressed in HEK‐293T cells, which were then immunoprecipitated using magnetic beads. The resulting lysates were separated by SDS‐PAGE and stained with Coomassie Brilliant Blue. Peptides were fractionated on an ultra‐high‐performance liquid chromatography system (EASY‐nLC 1200, Thermo Fisher Scientific) using a gradient of solvent A (0.1% formic acid, 2% acetonitrile in water) and solvent B (0.1% formic acid, 90% acetonitrile in water). Separated peptides were analyzed on a Q Exactive HF mass spectrometer (Thermo Fisher Scientific) equipped with a nanoelectrospray ionization source at 2.1 kV. Precursor and fragment ions were acquired at a resolution of 60 000 using the Orbitrap detector. Mass spectrometry data were processed with Proteome Discoverer 2.4 software for protein identification and quantification.

### 2.12 Co‐Immunoprecipitation (Co‐IP)

Cells were lysed in NP‐40 lysis buffer (Cat#: P0013F, Beyotime Biotechnology) supplemented with a protease inhibitor cocktail (Cat#: P1045, Beyotime Biotechnology) to prevent protein degradation. Half of the lysate supernatant was reserved as an input control, while the remainder was incubated with specific antibodies at 4°C overnight. Protein A/G agarose beads were then added and incubated for 2 h at room temperature to form immune complexes. The complexes were washed three times with 500 µL of lysis buffer, and bound proteins were analyzed by Western blotting. Protein concentrations of cell or tissue samples were determined using a BCA (Cat#: 23227, Thermo Fisher Scientific) protein assay kit following the manufacturer's instructions.

### 2.13 ELISA

The concentrations of Aβ40 and Aβ42 in brain tissue lysates were quantified using commercially available mouse ELISA kits (FineTest, Wuhan, China; Cat#: EM0863 and Cat#: EM0864) according to the manufacturer's instructions. The absorbance was measured using a microplate reader.

### 2.14 Adult Mouse Microglia Isolation and Flow Cytometry

Adult mouse brains were dissociated using the Brain Dissociation Kit (RWD, Shenzhen, China) according to the manufacturer's instructions. Briefly, mice were euthanized by cervical dislocation, and the brains were collected, minced into small fragments, and transferred into gentleMACS C Tubes containing the enzyme mixture. Tissue dissociation was performed using a gentleMACS Octo Dissociator with Heaters (Miltenyi Biotec, Cat#: 130‐096‐427) for 30 min. The resulting suspension was filtered through a 70‐µm cell strainer and then centrifuged to collect the cells. Cell debris and erythrocytes were subsequently removed according to the manufacturer's protocol. CD11b‐positive microglia were enriched by magnetic separation using CD11b MicroBeads (STEMCELL Technologies, Cat#: 18970) [27]. To evaluate the enrichment efficiency, the isolated cells were stained with FITC‐conjugated anti‐mouse CD11b antibody (BD Pharmingen, Cat#. 557396) in the dark and analyzed using a BD FACSVerse flow cytometer (BD Biosciences, USA).

### 2.15 Statistical Analysis

Data are presented as the mean ± standard error of the mean (SEM) from at least three independent experiments. The number of replicates (n) for each data point is indicated in the figure legends. Statistical analyses were performed using GraphPad Prism 10.0 (San Diego, CA). Differences between the two groups were assessed using a two‐tailed Student's *t‐*test. For comparisons involving more than two groups, one‐way analysis of variance (ANOVA) followed by Tukey's post hoc test was applied. A *p*‐value < 0.05 was considered statistically significant.

## 3 Results

### 3.1 OTUD7B Expression is Elevated in Brain Tissues of AD Patients and AD Mouse Models

**FIGURE 1:**
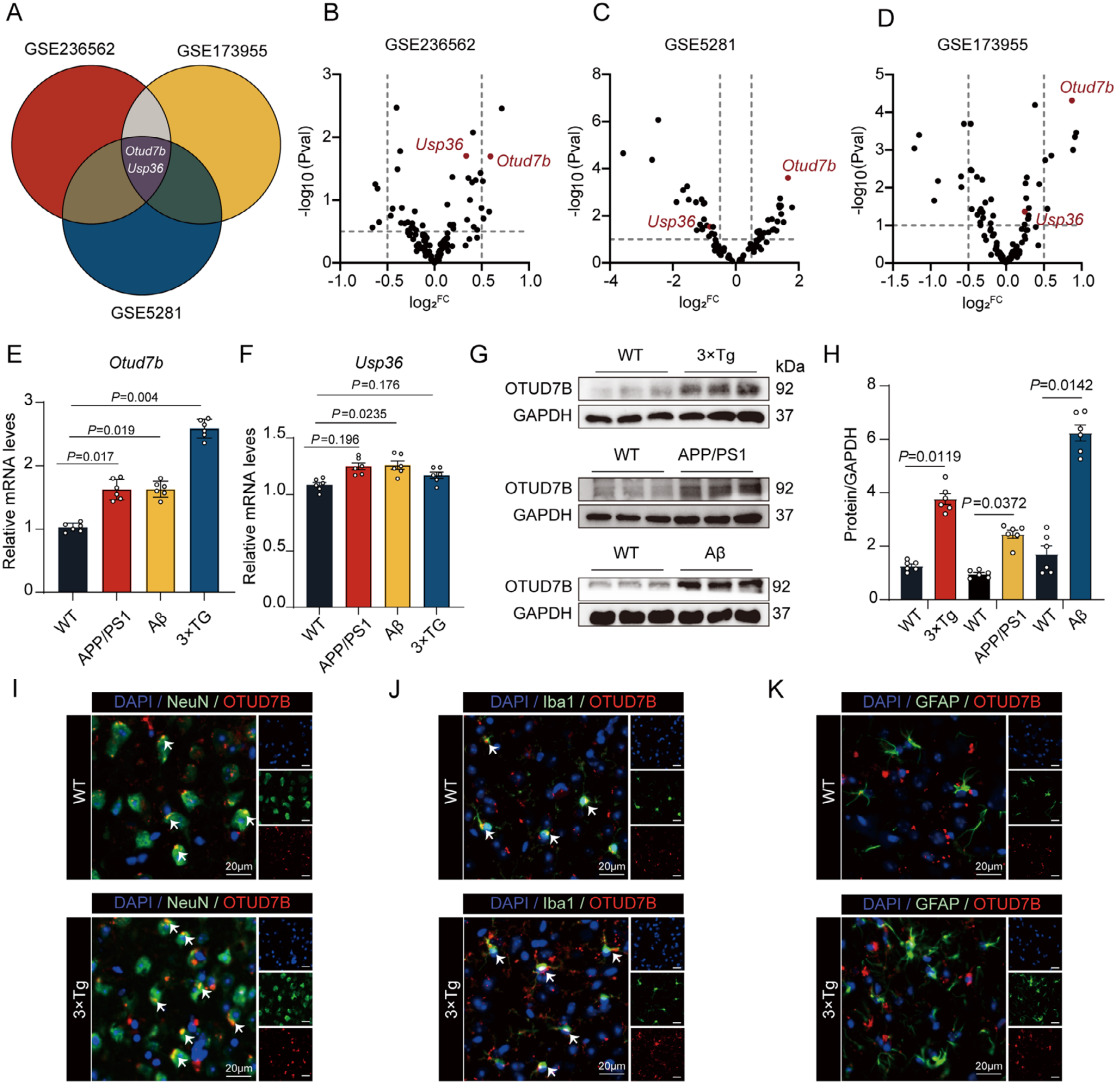
OTUD7B expression is elevated in AD patients and AD mouse models. (A) A Venn diagram highlighting enriched DUBs from three public RNA‐seq datasets (GSE236562, GSE5281, and GSE173955). (B–D) The DUB transcription profiles of control and AD patients were analyzed using volcano plots across different databases. (E,F) qPCR analysis of *Otud7b* and *Usp36* mRNA levels in APP/PS1, Aβ‐infusion mice, and 3×Tg AD models. (G) Western blot analysis of OTUD7B in APP/PS1, Aβ‐infusion mice, and 3×Tg AD models. GAPDH was used as the loading control. (H) Densitometric quantification of OTUD7B levels in panel G was presented. (I–K) Immunofluorescence staining localization analysis was conducted to determine the expression of OTUD7B in (I) NeuN, (J) Iba1, and (K) GFAP in the 3×Tg AD model mice. Statistical significance for all panels was determined by the unpaired Student's *t*‐test. Statistical data are presented as mean ± SEM (n = 6).

To investigate the potential role of deubiquitinases in AD, we analyzed differential expression profiles of all known deubiquitinases across three independent transcriptomic datasets: GSE236562, GSE5281, and GSE173955. By intersecting the DUBs across all three datasets, we identified two overlapping candidates (Figure 1A): *Otud7b* and *Usp36*. However, subsequent analysis revealed that *Usp36* was not among the significantly altered genes in the volcano plots (Figure 1B–D), and direct comparisons of normalized mRNA expression levels showed no statistically significant differences in AD patient brain tissues compared to controls (Figure S1A–C). In contrast, *Otud7b* consistently demonstrated significant differences across datasets, both in differential transcriptional analysis and in transcription‐level comparisons (Figure 1B–D and Figure S1D–F). Consistent with these findings, elevated *Otud7b* mRNA levels were also observed in multiple AD mouse models, including 3×Tg, APP/PS1, and Aβ‐infused wild‐type mice (Figure 1E), but no significant changes in *Usp36* were observed (Figure 1F). In addition, OTUD7B protein levels were markedly increased in the brain tissues of these AD mouse models (Figure 1G,H).

To explore the source of OTUD7B in the brain, we performed immunofluorescence staining on brain sections from 3×Tg mice. The results showed that OTUD7B was predominantly expressed in NeuN‐ (neuron marker) and Iba1 (microglia marker)‐positive cells (Figure 1I,J and Figure S1G–I), while a relatively weak signal was observed in GFAP (astrocyte marker)‐positive cells (Figure 1K). These results suggest that OTUD7B is upregulated in AD pathology and may function through neuron or microglia‐mediated pathways to contribute to AD pathogenesis.

### 3.2 Microglial OTUD7B Promotes Aβ‐induced Inflammation In Vitro

**FIGURE 2:**
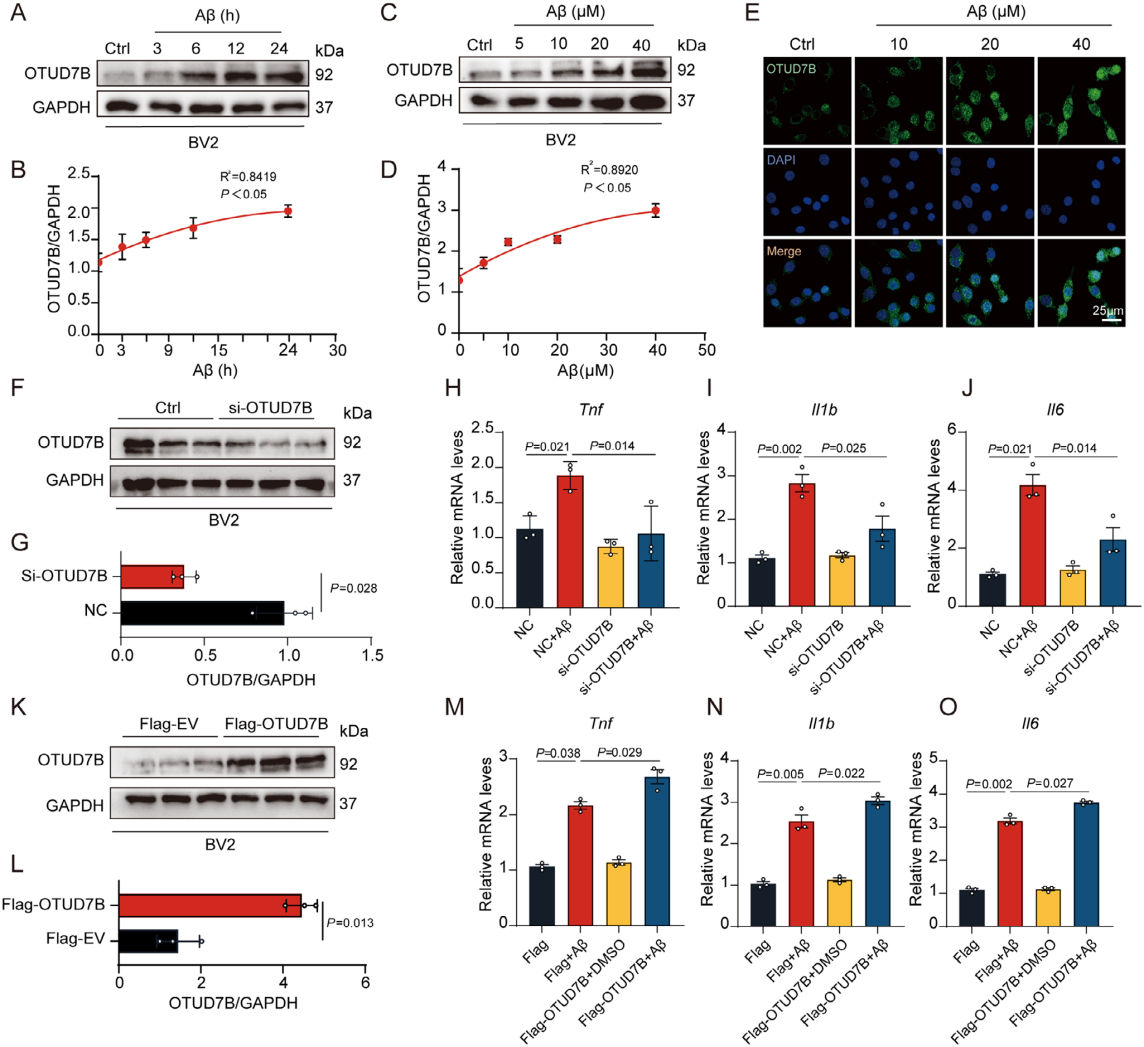
Microglial OTUD7B promotes Aβ‐induced inflammation in vitro. (A,B) BV2 cells were exposed to 20 µm of Aβ for 0, 3, 6, 12, and 24 h, respectively. The protein levels of OTUD7B were measured by Western blotting (A), and densitometric quantification is shown (B). GAPDH was used as the loading control. (C–E) BV2 cells were exposed to different concentrations of Aβ for 24 h. The protein level of OTUD7B was measured by Western blotting assays (C), densitometric quantification (D), and immunofluorescence staining images (E). (F,G) BV2 cells were transfected with si‐OTUD7B, while control cells were transfected with negative control siRNA. Western blotting assays were used to determine OTUD7B protein levels (F), and densitometric quantification is shown (G). GAPDH was used as the loading control. (H–J) BV2 cells were transfected with NC or si‐OTUD7B, followed by Aβ stimulation for 24 h. The mRNA levels of *Tnf* (H), *Il1b* (I), and *Il6* (J) were determined by RT‐qPCR. (K,L) BV2 cells were transfected with Flag‐OTUD7B, while control cells were transfected with Flag‐empty vector (Flag‐EV). Western blotting assay was used to determine OTUD7B protein levels (K), and densitometric quantification (L) is shown. The mRNA levels of *Tnf* (M), *Il1b* (N), and *Il6* (O) were determined by RT‐qPCR. Statistical analysis was performed using non‐linear regression analysis (B,D), unpaired Student's *t*‐test (G,L), and one‐way ANOVA followed by Tukey's multiple comparisons test (H–J, M–O). Statistical data are presented as mean ± SEM (n = 3).

To further determine whether OTUD7B exerts its effects primarily through neurons or microglia, we performed a series of temporal and dose‐dependent analyses of OTUD7B expression in BV2 microglial cells and SH‐SY5Y neuron cells. Western blotting combined with curve fitting revealed a stable, linear increase in OTUD7B expression over time or with increasing Aβ concentrations in BV2 cells (Figure 2A–D). These findings were further corroborated by immunofluorescence staining, which confirmed a dose‐dependent upregulation of OTUD7B in BV2 cells upon Aβ exposure (Figure 2E). In contrast, OTUD7B in SH‐SY5Y cells exhibited a transient and fluctuating response (Figure S2A–D).

To investigate the functional relevance of OTUD7B in these two cell types, we performed siRNA‐mediated knockdown in both BV2 (Figure 2F,G) and SH‐SY5Y (Figure S2E,F) cells. In BV2 cells, silencing of OTUD7B markedly attenuated Aβ‐induced upregulation of pro‐inflammatory cytokines, including *Tnf*, *Il1b*, and *Il6* (Figure 2H–J). However, this anti‐inflammatory effect was not observed in SH‐SY5Y cells following OTUD7B knockdown (Figure S2G–I). Furthermore, suppression of OTUD7B in SH‐SY5Y cells failed to restore the Aβ‐induced decrease in the Bcl‐2/Bax ratio (Figure S2J,K) and did not reduce neuronal apoptosis, as evidenced by flow cytometry (Figure S2L,M). Conversely, overexpression of OTUD7B (Flag‐OTUD7B) in BV2 cells significantly exacerbated Aβ‐induced inflammatory responses (Figure 2K–O). To further assess whether microglial OTUD7B affects neuronal survival in a non‐cell‐autonomous manner, we performed a conditioned medium transfer experiment (Figure S3A). Notably, exposure to conditioned medium from OTUD7B‐knockdown BV2 cells significantly alleviated the Aβ‐induced reduction in the Bcl‐2/Bax ratio in SH‐SY5Y cells (Figure S3B,C). In addition, flow cytometry analysis revealed a marked decrease in cellular apoptosis in SH‐SY5Y cells under this condition (Figure S3D,E).

Collectively, these findings suggest that although OTUD7B is expressed in both neurons and microglia, it primarily functions in microglial‐mediated inflammatory responses under Aβ stimulation, and its inhibition in microglia can exert protective effects on neurons.

### 3.3 Microglia‐Specific OTUD7B Knockdown Ameliorates Cognitive Deficits and Neuroinflammation in 3×Tg AD Mice

**FIGURE 3:**
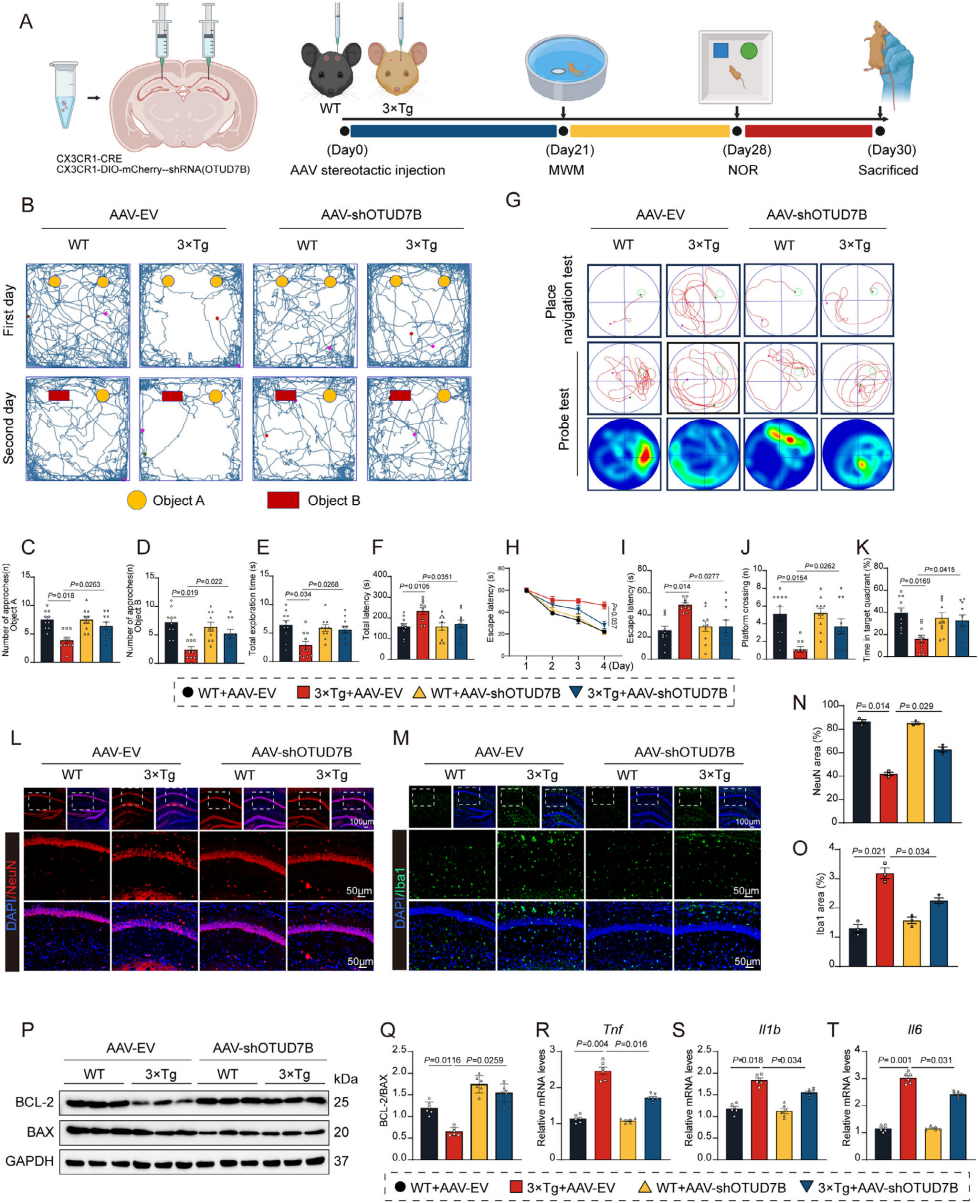
Microglia‐specific OTUD7B knockdown ameliorates cognitive deficits and neuroinflammation in 3×Tg AD Mice. (A) Schematic diagram of stereotactic hippocampal injection of AAV and subsequent behavioral experiment procedures and time points. (B) Representative images of the novel object recognition test. A total number of object A (C) and object B (D) approaches were explored on the second day. The total exploration time (E) to explore new object B and the total latency (F) of new object B approaches, n = 10. (G) Representative swimming trajectories in the place navigation test, along with swimming paths and corresponding heatmaps in the probe test. (H,I) Time required for mice to find the platform hidden under the water in the Place navigation test (Escape latency), n = 10. (J) The average number of times mice in each group crossed the platform within 60 s after the platform was removed (n = 10). (K) Time spent in the target quadrant where the platform is located after the platform is removed, n = 10. Representative immunofluorescence staining and quantification of NeuN (L,N) and Iba1 (M,O) in the hippocampus after sacrifice. Scale bar = 100 µm in the upper panels and 50 µm in the lower panels. (P) The protein levels of BCL‐2 and BAX in the brain were measured by Western blotting assays. GAPDH was used as the loading control. (Q) Densitometric quantification of BCL‐2/BAX levels in panel N was presented, n = 6. The mRNA levels of *Tnf* (R), *Il1b* (S), and *Il6* (T) were determined by RT‐qPCR (n = 6). Statistical significance across all panels was determined by one‐way ANOVA followed by Tukey's multiple‐comparison test. Statistical data are presented as mean ± SEM.

To investigate the role and mechanism of OTUD7B in AD, we generated a microglia‐specific OTUD7B conditional knockdown AAV9 (AAV‐shOTUD7B) and injected it into the hippocampus of age‐matched WT and 3×Tg AD mice (Figure 3A and Figure S4A,B). PCR and Western Blot on primary microglial cells confirmed successful knockdown of AAV‐OTUD7B in microglia (Figure S4C–H).

Three weeks after injection, cognitive function was assessed by novel object recognition (NOR) and Morris water maze (MWM) tests. In the NOR test, AAV‐shOTUD7B mice exhibited a significantly higher preference for the novel object compared to 3×Tg controls (Figure 3B–D), with a significantly increased discrimination index (Figure S4I), accompanied by increased total exploration time and decreased latency to explore the novel object (Figure 3E,F), indicating improved recognition memory. Consistently, MWM testing showed that AAV‐shOTUD7B mice had a significantly lower average escape latency compared to 3×Tg mice during acquisition trials (Figure 3G–I). In the probe trial after platform removal, AAV‐shOTUD7B mice demonstrated significantly increased platform crossing numbers and longer time spent in the target quadrant compared with 3×Tg mice (Figure 3J,K), indicating a partial improvement in spatial memory retention, although the performance remained below that of WT mice. Immunofluorescence analysis revealed that AAV‐shOTUD7B reduced neuronal loss in the hippocampus of 3×Tg mice and suppressed microglial activation as indicated by decreased Iba1 staining (Figure 3L–O), which is commonly used as an indirect indicator of microglial activation [28]. As expected, *Tmem119* transcription was reduced in the 3×Tg mice, while *Cd45* mRNA levels increased (Figure S5A,B), consistent with enhanced microglial activation and inflammatory responses. Knockdown of OTUD7B partially rescued these changes in the 3×Tg background, supporting the role of OTUD7B in promoting microglial inflammation. Minimal changes were observed in WT + AAV‐shOTUD7B mice, consistent with the low basal inflammatory state. Using ELISA analysis, we measured Aβ40 and Aβ42 levels in brain lysates from the AAV‐shOTUD7B 3×Tg mouse (Figure S5C,D). The results showed that OTUD7B knockdown did not significantly alter Aβ40 or Aβ42 levels, suggesting that OTUD7B does not directly regulate amyloid production or deposition. Moreover, consistent with our in vitro findings, AAV‐shOTUD7B reversed Aβ‐induced apoptosis and pro‐inflammatory cytokine production, as shown by restoration of the Bcl‐2/Bax ratio (Figure 3P,Q) and decreased mRNA levels of *Tnf*, *Il1b*, and *Il6* (Figure 3R–T).

In summary, these results indicate that microglia‐specific OTUD7B knockdown attenuated neuroinflammation and neuronal damage, without altering Aβ40/42 burden (Figure S5E), thereby partially improving cognitive performance in 3×Tg mice.

### 3.4 Microglial OTUD7B Deficiency Attenuated Aβ‐Induced Neuroinflammation and Cognitive Impairment in an Acute AD Model

**FIGURE 4:**
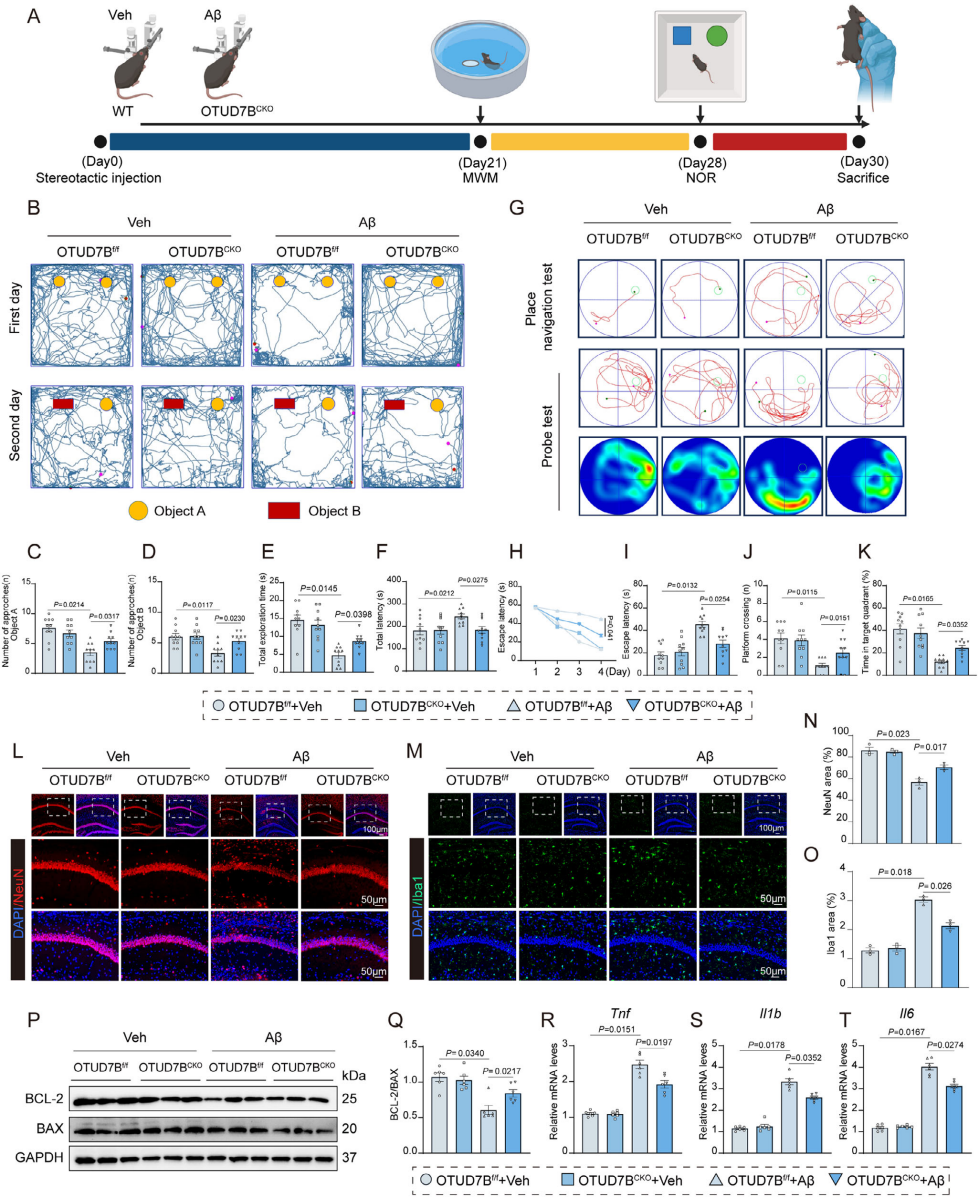
Microglial OTUD7B deficiency attenuates Aβ‐induced neuroinflammation and cognitive impairment in an acute AD model. (A) Injection of Aβ into the hippocampus of OTUD7B^CKO^ mice and subsequent experimental procedures. (B) Representative images of the novel object recognition test. A total number of object A (C) and object B (D) approaches were explored on the second day. The total exploration time (E) to explore new object B and the total latency (F) of new object B approaches, n = 10. (G) Representative swimming trajectories in the place navigation test, along with swimming paths and corresponding heatmaps in the probe test, are shown. (H,I) Time required for mice to find the platform hidden underwater in the Place navigation test (Escape latency). (J) The average number of times mice in each group crossed the platform within 60 s after removing the platform. (K) Time spent in the target quadrant where the platform is located after the platform is removed, n = 10. Representative immunofluorescence staining and quantification of NeuN (L,N) and Iba1 (M,O) in the hippocampus after sacrifice, Scale bar = 100 µm in the upper panels and 50 µm in the lower panels. (P) The protein levels of BCL‐2 and BAX in the brain were measured by Western blotting assays. GAPDH was used as the loading control. (Q) Densitometric quantification of BCL‐2/BAX levels in panel N was presented (n = 6). The mRNA levels of *Tnf* (R), *Il1b* (S), and *Il6* (T) were determined by RT‐qPCR (n = 6). Statistical significance across all panels was determined using one‐way ANOVA followed by Tukey's multiple‐comparison test. Statistical data are presented as mean ± SEM.

To further elucidate the role of OTUD7B in AD pathogenesis, we established microglia‐specific OTUD7B conditional knockout (OTUD7B^CKO^) mice and verified the selective downregulation of OTUD7B expression in microglial cells within the brain (Figure S4J–M). To induce acute neuroinflammation and neuronal damage, we performed stereotactic injection of Aβ (10 µg per mouse) into the hippocampus. OTUD7B^f/f^ mice receiving equivalent volumes of vehicle (Veh) served as the controls. Three weeks post‐injection, a battery of behavioral tests was conducted to assess cognitive performance (Figure 4A).

Consistent with findings in the 3×Tg AD model, Aβ infusion reduced total exploration time and increased the latency to explore the novel object, whereas OTUD7B^CKO^ mice exhibited increased total exploration time and decreased latency compared with Aβ‐treated OTUD7B^f/f^ mice (Figure 4B–F). In the MWM test, Aβ infusion led to a marked prolongation of escape latency in OTUD7B^f/f^ mice, whereas OTUD7B^CKO^ mice demonstrated a significantly shorter average escape latency across the training days (Figure 4G–I). During the probe trial, OTUD7B^CKO^ mice displayed increased platform crossings and spent more time in the target quadrant than Aβ‐treated OTUD7B^f/f^ mice, indicating a partial improvement in spatial memory retention (Figure 4J,K). Histological analysis revealed that OTUD7B^CKO^ alleviated Aβ‐induced neuronal loss and reduced microglial accumulation, as indicated by decreased Iba1 in the hippocampus (Figure 4L–O). In line with this, OTUD7B^CKO^ also suppressed neuronal apoptosis (Figure 4P,Q) and significantly reduced the expression of pro‐inflammatory cytokines, including *Tnf*, *Il1b*, and *Il6* (Figure 4R–T).

Together, these data indicate that OTUD7B in microglia contributes to Aβ‐induced neuroinflammatory responses and cognitive dysfunction, and that selective deletion of OTUD7B in microglia partially ameliorates Aβ‐induced pathological and behavioral impairments in AD‐related pathology. Notably, because the discrimination index on the NOR test did not change significantly, the NOR findings should be interpreted as reflecting changes in exploratory activity rather than definitive improvements in recognition memory.

### 3.5 OTUD7B Directly Interacts With STAT3

**FIGURE 5:**
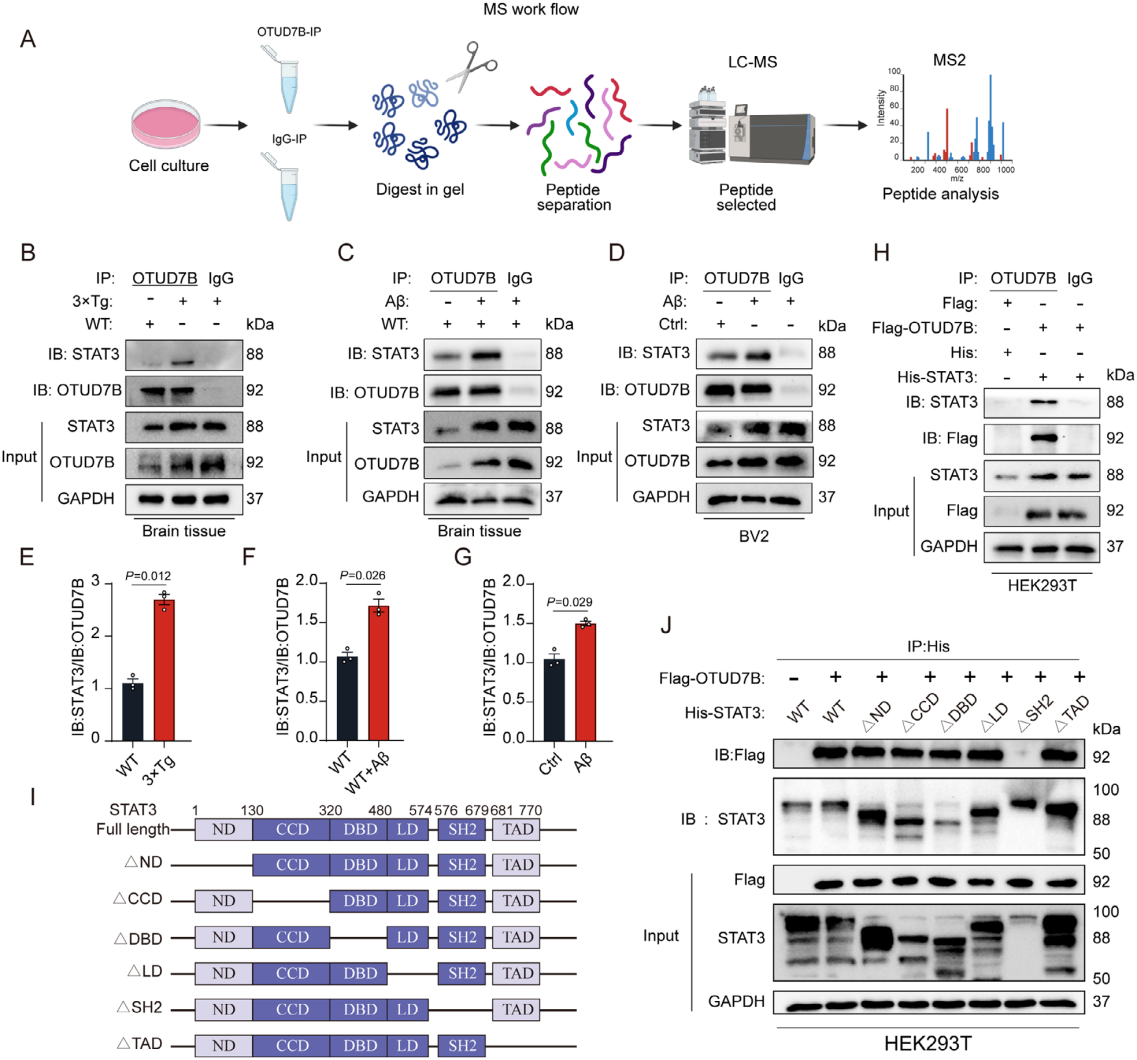
OTUD7B directly interacts with STAT3. (A) Schematic illustration of OTUD7B‐IP and IgG‐IP in HET293T cells followed by proteomic screening. (B–D) Co‐IP of endogenous OTUD7B and STAT3 in 3×Tg brain tissues (B), WT+Aβ brain tissues (C), and BV2 cells following Aβ stimulation (D). (E–G) Densitometric quantification of STAT3/OTUD7B levels in panel B–D was presented. (H) Co‐IP of OTUD7B and STAT3 in HEK293T cells co‐transfected with Flag‐OTUD7B and His‐STAT3 plasmids. (I) A schematic representation of the STAT3 domain deletion construct used in (J). Co‐IP of WT‐STAT3, mut‐STAT3, and OTUD7B in HEK293T cells co‐transfected with overexpression plasmids of His‐WT‐STAT3, His‐mut‐STAT3, or Flag‐OTUD7B. Statistical significance for all panels was determined by the unpaired Student's *t*‐test. Statistical data are presented as mean ± SEM (n = 3).

To elucidate the molecular mechanism by which OTUD7B functions in AD and to identify its potential substrate, we first performed co‐immunoprecipitation (Co‐IP) followed by LC‐MS/MS analysis (Figure 5A). Given that DUBs often regulate biological processes by modulating the degradation or activity of their substrates [29], we prioritized candidate interactors by considering not only enrichment in the MS analysis but also their biological relevance to microglial inflammatory signaling. Although KRT18 displayed a relatively high fold enrichment in the proteomic dataset (Figure S6A), it is primarily known as an epithelial intermediate filament protein and has limited evidence supporting a role in microglial function or AD‐related neuroinflammation. In contrast, STAT3 is a well‐established transcription factor critically involved in microglial activation and inflammatory gene regulation, and its corresponding MS/MS fragmentation spectrum is shown in Figure S6B. These results led us to hypothesize that STAT3 may serve as an OTUD7B substrate in the brain and mediate its functional role in AD pathology.

To validate this hypothesis, we confirmed the endogenous interaction between OTUD7B and STAT3 in the brains of 3×Tg and Aβ‐infused mice, and in Aβ‐treated BV2 cells using Co‐IP (Figure 5B–G). Interaction between exogenous OTUD7B and STAT3 was also observed in HEK293T cells transfected with OTUD7B and STAT3 overexpression plasmids (Figure 5H). To further delineate the binding interface within this interaction, a series of deletion mutants of STAT3 were constructed based on its predicted domain architecture (Figure 5I). Co‐IP analysis revealed that the SH2 (Src Homology 2) domain of STAT3 was involved in its interaction with OTUD7B (Figure 5J).

### 3.6 OTUD7B Stabilizes STAT3 by Removing K48‐Linked Ubiquitin Chains

**FIGURE 6:**
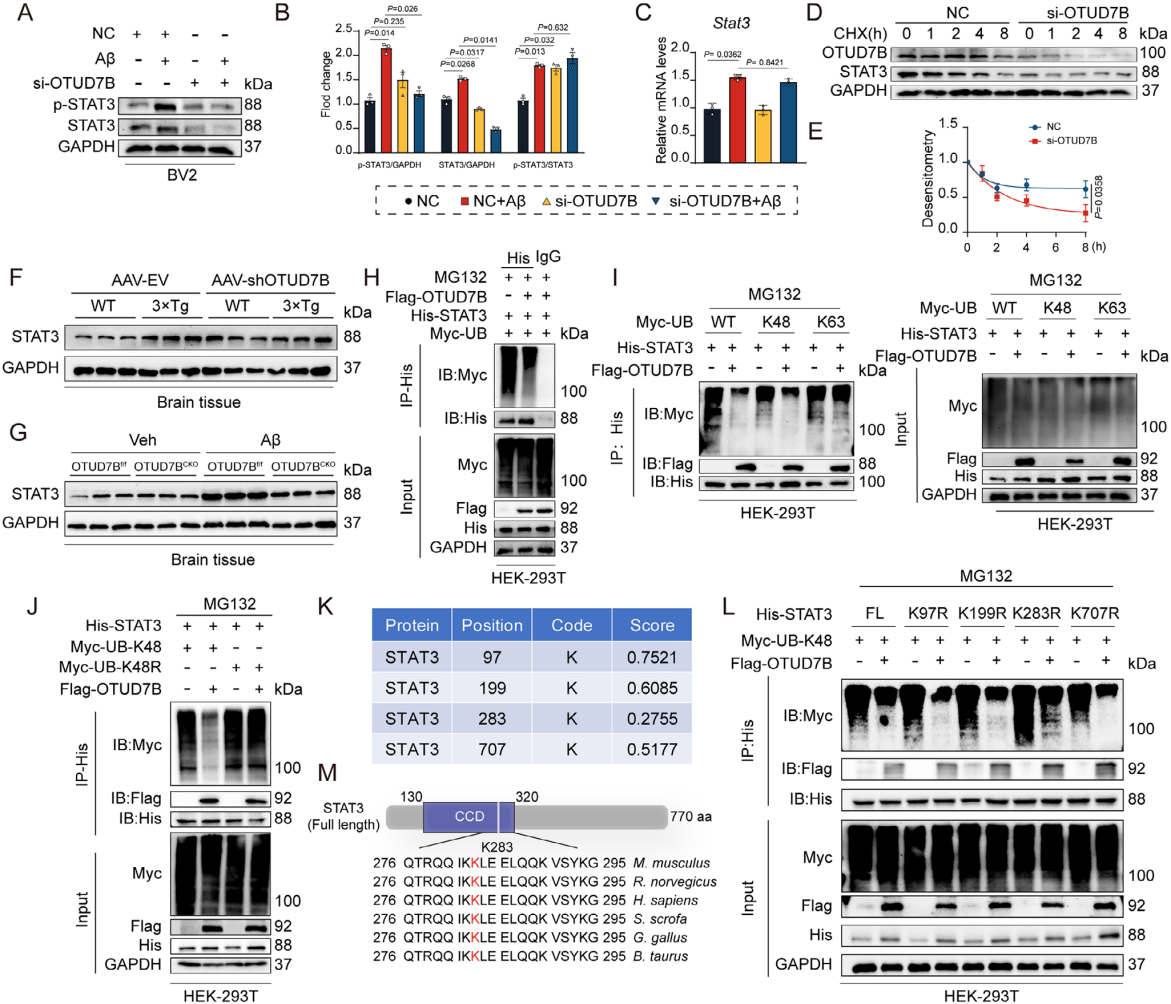
OTUD7B stabilizes STAT3 by removing K48‐linked ubiquitin chains. (A,B) Representative Western blotting images of p‐STAT3 and STAT3 (A), and the densitometric quantification are shown (B). (C) Real‐time qPCR analysis of mRNA levels of *Stat3* in Aβ‐treated BV2 cells after transfection with si‐OTUD7B. (D) Representative Western blotting images of STAT3 in HEK‐293T cells transfected with si‐OTUD7B, followed by a CHX pulse‐chase assay. (E) Densitometric quantification of panel D is shown. (F,G) Representative Western blotting images of STAT3 in brain tissues of 3×Tg injected with AAV‐shOTUD7B (F) and Aβ‐stimulated OTUD7B^CKO^ mice (G). (H) Co‐IP of STAT3 in HEK293T cells co‐transfected with overexpression plasmids of His‐STAT3, Flag‐OTUD7B, and Myc‐Ub. (I) Co‐IP of STAT3 in HEK‐293T cells co‐transfected with plasmids of His‐STAT3, Flag‐OTUD7B, Myc‐Ub, Myc‐Ub‐K48, and Myc‐Ub‐K63. (J) Co‐IP of STAT3 in HEK‐293T cells co‐transfected with overexpression plasmids of His‐STAT3, Flag‐OTUD7B, Myc‐Ub‐K48, and Myc‐Ub‐K48R. (K) GPS‐Uber analysis showed potential OTUD7B‐regulated ubiquitination of STAT3 lysine residues. (L) Co‐IP of STAT3 in HEK293T cells that were co‐transfected with overexpression plasmids encoding Flag‐OTUD7B, His‐STAT3‐full‐length (FL), His‐STAT3‐K97R, His‐STAT3‐K199R, His‐STAT3‐K283R, His‐STAT3‐K707R, and Myc‐Ub‐K48. (M) Schematic representation of the sequence of STAT3‐K283 across various species. Statistical significance across all panels was determined using one‐way ANOVA followed by Tukey's multiple‐comparison test. Statistical data are presented as mean ± SEM (n = 3).

Ubiquitination typically serves as a signal for proteasomal degradation of proteins, whereas deubiquitination can reduce protein turnover, thereby enhancing protein stability [30]. We first observed that genetic deletion of OTUD7B significantly reduced both total and phosphorylated STAT3 levels in BV2 cells (Figure 6A,B), while the mRNA levels of STAT3 remained unchanged (Figure 6C), suggesting that OTUD7B regulated STAT3 post‐transcriptionally. In contrast, overexpression of OTUD7B (Flag‐OTUD7B) led to increased STAT3 levels (Figure S7A,B) and was accompanied by an increase in p‐STAT3 levels, normalization of p‐STAT3 to total STAT3 showed that OTUD7B manipulation did not significantly alter the p‐STAT3/STAT3 ratio under Aβ stimulation in either the knockdown or overexpression models (Figure 6B and Figure S7B). These findings indicate that OTUD7B primarily regulates STAT3 protein abundance rather than its phosphorylation efficiency. Notably, *Stat3* mRNA levels showed a modest change compared with the Flag + Aβ group (Figure S7C), suggesting that OTUD7B primarily regulates STAT3 at the post‐transcriptional level. To further confirm this, we blocked protein synthesis with cycloheximide (CHX) and monitored STAT3 protein levels. As shown in Figure 6D,E, OTUD7B deficiency accelerated the degradation of STAT3 in a time‐dependent manner, whereas Flag‐OTUD7B delayed its degradation (Figure S7D,E). In vivo, OTUD7B^CKO^ also reduced STAT3 levels in both 3×Tg and Aβ‐infused mouse models (Figure 6F,G and Figure S7F,G), further indicating that OTUD7B promoted STAT3 stability. Consistently, OTUD7B deficiency in 3×Tg mice led to a reduction in total STAT3 levels, accompanied by a corresponding decrease in p‐STAT3 levels in vivo (Figure S7H–K). These results further support our conclusion that OTUD7B regulates STAT3 protein stability, thereby influencing p‐STAT3 levels. To determine the degradation pathway involved, we treated cells with the proteasome inhibitor MG132 or the lysosome inhibitor chloroquine (CQ). We found that MG132 further enhanced STAT3 accumulation in Flag‐OTUD7B cells (Figure S8A,B), whereas CQ had no effect (Figure S8C,D). Further analysis showed that STAT3 ubiquitination was significantly reduced in the presence of OTUD7B, and OTUD7B significantly increased STAT3 protein levels by promoting its deubiquitination (Figure 6H). These data indicate that OTUD7B positively regulates the post‐translational stability of STAT3, which may be highly correlated with ubiquitination modification.

Studies have shown that DUBs inhibit the degradation of substrate proteins by cleaving K48‐ or K63‐linked Ub chains on these proteins [31]. Thus, we performed deubiquitination assays using specific ubiquitin mutants. OTUD7B preferentially removed K48‐linked ubiquitin chains from STAT3 (Figure 6I). Moreover, the K48R mutant but not the K63R, abolished the deubiquitination effect of OTUD7B (Figure 6J and Figure S9A), confirming that OTUD7B specifically targeted K48‐linked polyubiquitin chains. To identify the precise lysine residue on STAT3 targeted by OTUD7B, we predicted and generated four lysine‐to‐arginine point mutants of STAT3: K97R, K199R, K283R, and K707R (Figure 6K). Among these, only the K283R mutant blocked OTUD7B's ability to reduce STAT3 ubiquitination and abrogated OTUD7B's stabilizing effect on STAT3 protein levels (Figure 6L and Figure S9B), suggesting K283 as the key deubiquitination site. Notably, K283 is highly conserved across multiple species (Figure 6M). Furthermore, expression of the K283R mutant reversed the pro‐inflammatory effects associated with Flag‐OTUD7B in BV2 cells (Figure S9C–E).

Collectively, these findings demonstrate that OTUD7B stabilizes STAT3 by removing K48‐linked ubiquitin chains specifically at K283, thereby preventing its proteasomal degradation and modulating downstream inflammatory signaling.

### 3.7 OTUD7B Deficiency in Microglia Suppresses the STAT3‐NF‐κB Signaling Pathway to Alleviate Neuroinflammation

**FIGURE 7:**
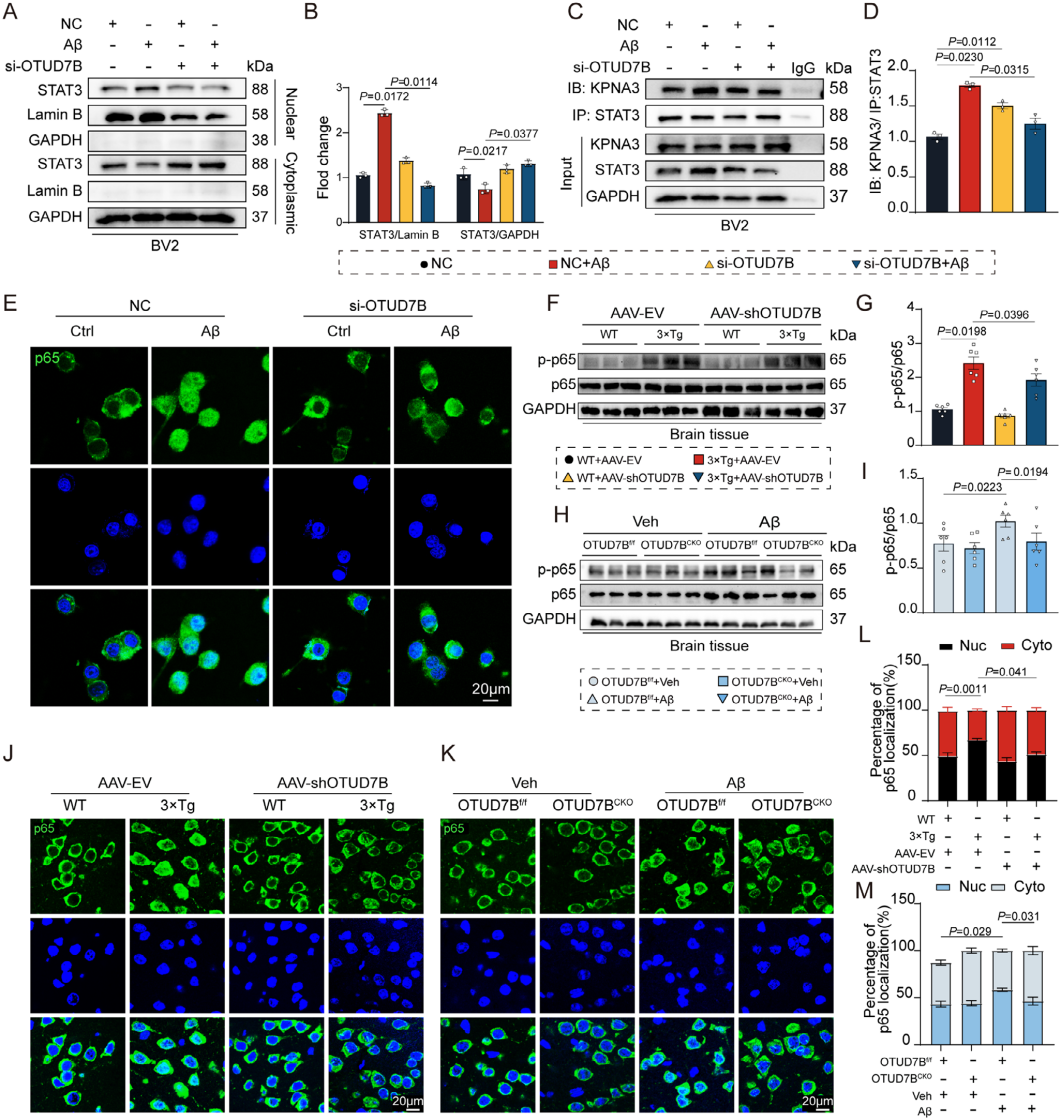
OTUD7B deficiency suppresses STAT3‐NF‐κB p65 signaling to alleviate neuroinflammation. (A) BV2 cells were transfected with NC or si‐OTUD7B, respectively. Cells were then challenged with Aβ for 24 h. Levels of STAT3 in nuclear and cytosolic fractions were determined by immunoblotting. Lamin B and GAPDH were used as loading controls. (B) Densitometric quantification of STAT3/Lamin B and STAT3/GAPDH levels in panel A was presented (n = 3). (C) BV2 cells were treated as indicated in Panel A. STAT3 IP was performed, and levels of KPNA3 (importin‐α3) were detected by immunoblotting. IgG IP was used as the loading control. (D) Densitometric quantification of KPNA3/STAT3 level in panel C was presented (n = 3). (E) BV2 cells were treated as indicated in Panel A. Representative images of NF‐κB p65 (green) immunofluorescence staining in the BV2 cells. Scale bar = 20 µm. (F–I) Representative Western blot analysis and densitometric quantification of p‐p65 and p65 in brain tissue of 3×Tg injected with AAV‐shOTUD7B (F,G) and Aβ‐treated OTUD7B^CKO^ mice (H‐I). (J,K) Representative immunofluorescence staining of p65 in brain tissues of 3×Tg injected with AAV‐OTUD7B (J) and Aβ‐induced OTUD7B^CKO^ mice (K). (L,M) The percentage of nuclear and cytoplasmic p65 was quantified in panel J (L) and K (M) (n = 6). Statistical significance was assessed using two‐way ANOVA followed by Tukey's multiple‐comparison test (L,M); others were assessed using one‐way ANOVA followed by Tukey's multiple‐comparison test. Statistical data are presented as mean ± SEM.

STAT3 activation requires phosphorylation and subsequent nuclear import [32, 33]. To further investigate the downstream effects of OTUD7B‐mediated STAT3 stabilization, we examined STAT3 activation and nuclear translocation. By separating nuclear and cytoplasmic fractions, we found that si‐OTUD7B significantly reduced STAT3 nuclear translocation (Figure 7A,B), whereas Flag‐OTUD7B enhanced STAT3 nuclear localization (Figure S10A,B). Since KPNA3 is a key nuclear transport receptor that facilitates STAT3 nuclear import [34, 35], we hypothesized that OTUD7B may promote STAT3 nuclear translocation by stabilizing STAT3, thereby increasing the availability of phosphorylated STAT3 to associate with KPNA3. Co‐IP assays showed that Aβ stimulation increased STAT3‐KPNA3 binding, but this interaction was suppressed lacking OTUD7B (Figure 7C,D) and enhanced upon Flag‐OTUD7B in BV2 cells (Figure S10C,D).

Once translocated to the nucleus, STAT3 is known to interact with the NF‐κB p65 subunit to promote transcription of pro‐inflammatory genes, thereby amplifying neuroinflammation [36]. Given that sustained p65 activation contributes to AD pathology [37, 38], we assessed p65 phosphorylation and nuclear localization both in vitro and in vivo. Our data show that si‐OTUD7B markedly inhibited Aβ‐induced p65 nuclear translocation in BV2 cells (Figure 7E and Figure S10E). In contrast, overexpression of OTUD7B did not significantly alter p65 nuclear localization in response to Aβ stimulation (Figure S10F,G), likely due to Aβ’s strong effect. Consistently, AAV‐shOTUD7B reduced p65 phosphorylation and nuclear translocation in brain tissues of 3×Tg mice, with similar effects observed in Aβ‐infused OTUD7B^CKO^ mice (Figure 7F–M). To further clarify the transcriptional regulatory role of STAT3 in this process, we performed chromatin immunoprecipitation followed by quantitative PCR (ChIP‐qPCR) to examine STAT3 binding at the promoter regions of key pro‐inflammatory cytokine genes, including *Tnf*, *Il1b*, and *Il6*. The results demonstrate that STAT3 binding to these promoters significantly decreased in si‐OTUD7B‐transfected HEK293T cells (Figure S10H).

In summary, these results demonstrate that OTUD7B deficiency in microglia disrupts STAT3 nuclear translocation and the transcriptional activation of pro‐inflammatory cytokines, which, in turn, suppress NF‐κB p65 signaling and reduce neuroinflammatory responses.

### 3.8 OTUD7B Enhances STAT3‐Dependent Aβ‐Induced Microglial Neuroinflammation

**FIGURE 8:**
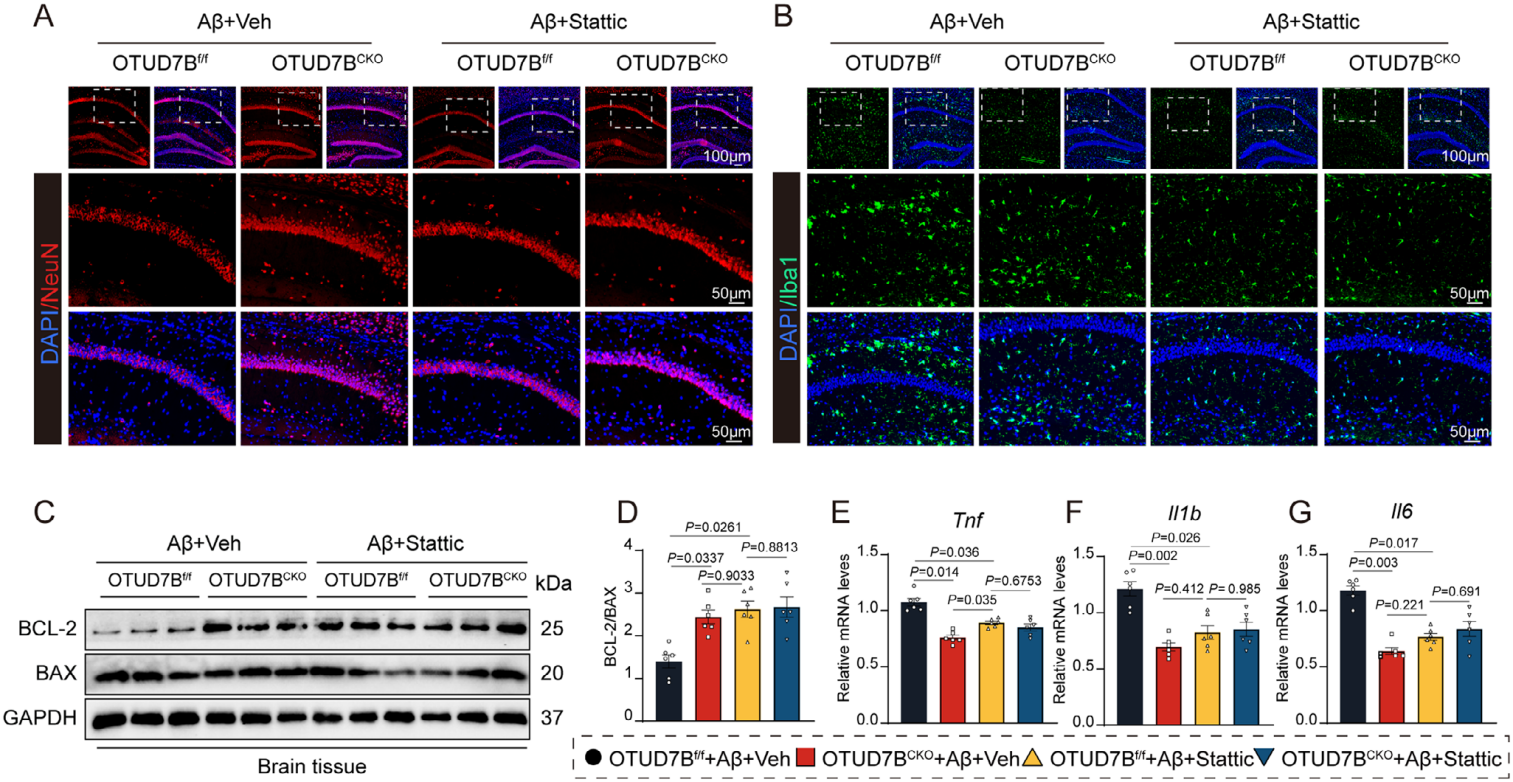
OTUD7B enhances STAT3‐dependent Aβ‐induced microglial neuroinflammation. To verify the effect of OTUD7B on STAT3, the STAT3 inhibitor Stattic (10 mg/kg, gavage) or vehicle [0.5% O­carboxymethylcellulose (CMC)‐Na and 0.25% Tween 80] was administered every 3 days via oral gavage. Healthy OTUD7B^CKO^ mice and OTUD7B^f/f^ mice were injected with Aβ or DMSO via stereotactic injection. Representative immunofluorescence staining of NeuN (A) and Iba1 (B) in the hippocampus after sacrifice, scale bar = 100 µm in the upper panels and 50 µm in the lower panels. (C) The protein levels of BCL‐2 and BAX in the brain were measured by Western blotting. GAPDH was used as the loading control. (D) Densitometric quantification of BCL‐2/BAX levels in panel C was presented (n = 6). The mRNA levels of *Tnf* (E), *Il1b* (F), and *Il6*(G) were determined by RT‐qPCR (n = 6). Statistical significance for all panels was determined using two‐way ANOVA followed by Tukey's multiple‐comparison test. Statistical data are presented as mean ± SEM.

To assess whether the effects of OTUD7B are mediated via STAT3, Aβ‐infused OTUD7B^f/f^ and OTUD7B^CKO^ mice were treated with stattic, a selective STAT3 inhibitor [39]. Fluorescence staining results show that OTUD7B deletion in microglia alone significantly attenuated Aβ‐induced neuronal loss and microglial activation (Figure 8A,B and Figure S11). Treatment with stattic similarly reduced neuronal damage and microglial reactivity in Aβ‐infused OTUD7B^f/f^ mice. However, OTUD7B^CKO^ animals did not exhibit further improvement beyond that achieved by stattic treatment in OTUD7B^f/f^ controls (Figure 8A,B and Figure S11). Specifically, stattic treatment did not further reduce Aβ‐induced apoptosis in brain tissues of OTUD7B^CKO^ mice compared with those of stattic‐treated OTUD7B^f/f^ mice (Figure 8C,D). Likewise, analysis of inflammatory gene transcription in brain tissues showed that inhibition of STAT3 by stattic diminished Aβ‐induced cytokine transcription, but OTUD7B deletion in microglia did not augment this effect (Figure 8E–G).

Together, these results indicated that the protective effect conferred by OTUD7B deficiency in microglia largely depended on STAT3 activity. Pharmacological blockade of STAT3 recapitulated the anti‐inflammatory and neuroprotective phenotype of OTUD7B deletion in microglia and eliminated any additional benefit of OTUD7B loss. Thus, the data supported a functional OTUD7B‐STAT3 signaling axis in the regulation of Aβ‐induced neuroinflammation.

## 4 Discussion

This study identifies for the first time that the deubiquitinase, OTUD7B, is a critical regulator of microglial inflammation in AD. OTUD7B was significantly upregulated in AD mouse models and human‐related databases, with high levels observed in both microglia and neurons. However, functional analyses revealed that OTUD7B depletion alleviated microglial hyperactivation and inflammatory responses, while its loss in neurons did not affect survival. Mechanistically, OTUD7B exerted its effect through deubiquitinating activity, specifically removing K48‐linked ubiquitin chains from lysine 283 of STAT3, thereby stabilizing STAT3 and enhancing the transcription of downstream inflammatory mediators. Collectively, these findings clarify the role of OTUD7B in AD‐associated neuroinflammation and underscore its potential as a therapeutic target.

Neurodegenerative diseases are characterized by the progressive loss of neuronal structure and function, often accompanied by the accumulation of misfolded and abnormally aggregated proteins. Dysregulation of DUBs, including mutations or loss of function, can destabilize substrate proteins and impair the clearance of misfolded proteins, ultimately leading to cell death, accelerated neuronal loss, developmental abnormalities, and neurodegeneration [40, 41]. Given their pivotal regulatory roles, DUBs have emerged as promising drug targets, and initial progress has been made in developing highly selective inhibitors [42, 43].

Based on structural and functional features, DUBs are categorized into seven families: ubiquitin‐specific proteases (USPs), ubiquitin carboxyl‐terminal hydrolases (UCHs), ovarian tumor proteases (OTUs), Machado‐Joseph disease proteases (MJDs), JAMM/MPN domain‐associated metallopeptidases (JAMMs), zinc finger‐containing ubiquitin peptidases (ZUP1s), and the MINDY family. To date, most studies in neurodegenerative disease have focused on USPs, such as USP7 [44], USP11 [45], USP14 [46], and USP30 [47], though recent work has implicated OTU family members such as OTUD3 [48] and OTUD5 [49]. However, these studies largely address the neuronal regulatory functions of DUBs, whereas increasing evidence highlights the central role of microglia‐mediated neuroinflammation in AD progression [50, 51]. Overactivated microglia may aberrantly label healthy neurons and engulf synapses, thereby driving synaptic degeneration [52, 53]. Persistent microglial migration and chronic inflammation further deteriorate the brain microenvironment, leading to neuronal damage [54]. Several DUBs implicated in inflammation have recently been reported, including USP18 [14] and USP19 [55]. Our previous work demonstrated that OTUD1 regulates microglial inflammation via C/EBPβ deubiquitination [56], while YOD1 maintains microglial homeostasis by deubiquitinating MYH9, thereby contributing to AD pathogenesis [57].

In this study, our immunofluorescence analysis, together with the reanalysis of a public RNA‐seq dataset, supports increased OTUD7B expression in microglia in AD models. Notably, although OTUD7B has been reported to be expressed in multiple cell types, its expression pattern appears to be highly dependent on cell type and disease context. For example, in acute inflammatory conditions such as experimental autoimmune encephalomyelitis, OTUD7B expression has been observed in astrocytes [58], whereas in the context of AD, which is characterized by chronic neuroinflammation, our data support a preferential enrichment in microglia. These differences likely reflect disease‐specific inflammatory environments, cellular activation states, and transcriptional regulatory mechanisms. OTUD7B is a multi‐specific DUB that regulates both K48‐and K63‐linked ubiquitin chains, with reported functions in cell proliferation, immunity, and inflammation [59]. K48‐linked chains typically mediate proteasomal degradation, whereas K63‐linked ubiquitination promotes protein activity or interactions [60]. Previous studies showed that OTUD7B enhances PIK3C degradation via K48 ubiquitination [61] and removes K63‐linked chains from IRF3 at K7, activating SQSTM1/p62‐dependent signaling [21]. Our findings expand this knowledge by identifying OTUD7B as a regulator of STAT3 stability. Specifically, OTUD7B removes K48‐linked ubiquitin chains from STAT3 at lysine 283, thereby preventing degradation and enhancing transcription of inflammatory mediators.

STAT3 is a well‐established mediator of AD pathology. Inhibition of STAT3‐driven astrocyte proliferation ameliorates AD‐like pathology in mice, and STAT3 is considered a central regulator of AD risk gene networks [62, 63]. Downregulation of ERK‐STAT3 signaling induces astrocytic neprilysin release, reducing Aβ burden [64]. Conversely, overactivation of STAT3 promotes neuroinflammation and behavioral impairments, making it an attractive therapeutic target [65, 66, 67]. However, targeting STAT3 for AD therapy still faces significant challenges. The main obstacle is that STAT3 is widely expressed across multiple tissues and participates in diverse physiological and pathological processes [39, 68]. Recent studies have indicated that microglial STAT3 is a key signaling driver of AD‐related neuroinflammation, and its overactivation exacerbates the inflammatory response and accelerates disease progression [69, 70]. This is consistent with our findings: under steady‐state conditions, OTUD7B deficiency only slightly downregulated STAT3, with no significant differences observed in animal models, suggesting limited regulatory effects or compensation by other pathways. By contrast, under Aβ stimulation, OTUD7B deficiency markedly decreased STAT3 expression, implying a negative regulatory role through specific stress‐responsive signaling mechanisms. Thus, targeting the OTUD7B‐STAT3 axis may enable microglia‐specific STAT3 inhibition while minimizing systemic side effects. Notably, we observed that the basal p‐STAT3/STAT3 ratio upon OTUD7B manipulation varied depending on the transfection approach (Figure 6B and Figure S7B), likely reflecting different degrees of transfection‐induced cellular stress and compensatory responses. Nevertheless, upon Aβ stimulation, OTUD7B deficiency or overexpression consistently modulated STAT3 stability across all experimental systems.

Currently, specific small‐molecule inhibitors of OTUD7B remain limited. In January 2025, a research team at the University of North Carolina reported 7Bi, the first OTUD7B inhibitor identified through AI‐assisted screening [71]. 7Bi inhibits OTUD7B's deubiquitination activity and blocks Akt signaling, showing strong antitumor effects in non‐small cell lung cancer and leukemia cells [71]. However, its selectivity has not yet been systematically evaluated, and potential off‐target effects on other DUB family members remain unclear. The development of highly specific OTUD7B inhibitors, therefore, remains an urgent challenge. Given that OTUD7B regulates protein stability and signaling via K48/K63 ubiquitin chains, the interaction between its OTU domain and substrates represents a promising entry point for drug design. Future studies may focus on protein‐protein interaction (PPI) inhibitors, including small molecules or peptides targeting the OTUD7B‐substrate interface, thereby enabling precise functional modulation. Such strategies would not only deepen mechanistic understanding of OTUD7B but also provide potential therapeutic interventions for AD and related diseases.

Despite these advances, our study has certain limitations. First, the results were primarily derived from cell lines and mouse models, and further validation in the human nervous system is required. Species differences may lead to discrepancies in AD pathogenesis and neuroinflammation between mice and humans. In addition, only male mice were used in this study to reduce potential variability associated with sex differences. Therefore, whether the observed effects of OTUD7B are sex‐dependent warrants further investigation in future studies. Second, our work focused on OTUD7B's regulation of STAT3, but it is likely that OTUD7B targets multiple substrates. Its roles in inflammation, apoptosis, and signal transduction may therefore involve additional mechanisms. Future studies combining ubiquitination substrate screening with proteomic approaches will be essential to systematically define the OTUD7B substrate repertoire and more comprehensively elucidate its roles in neuroinflammation and neuroprotection. In addition, although our microglia‐specific knockout strategy was designed to delete OTUD7B primarily in microglia, immunofluorescence data indicated that a proportion of OTUD7B‐positive cells were Iba1‐negative, suggesting expression of OTUD7B in non‐microglial cell types. Future studies employing more precise cell‐type‐specific labeling will be needed to more rigorously dissect the microglia contribution of OTUD7B to AD pathology. Third, although microglial activation is closely associated with Aβ pathology in AD, the present study primarily focused on microglia‐mediated inflammatory mechanisms. While soluble Aβ levels were evaluated by ELISA, we did not comprehensively assess Aβ plaque pathology or the spatial relationship between OTUD7B‐positive microglia and Aβ deposits, nor did we evaluate Tau pathology. The potential contribution of OTUD7B to these pathological features warrants further investigation. Fourth, although the NOR test was employed to assess recognition memory across experimental groups, the discrimination index did not reach statistical significance among groups in the OTUD7B^CKO^ mouse model (Figure 4B–D), and therefore, conclusions regarding recognition memory in this model are limited to changes in exploratory activity. The lack of significant differences in the discrimination index may be partly attributed to the relatively small sample size or inherent variability in spontaneous exploratory behavior. Future studies with larger cohorts or complementary behavioral paradigms, would provide more robust evidence for the effects of OTUD7B on recognition memory.

## 5 Conclusion

This study demonstrates that OTUD7B enhances STAT3 signaling by removing K48‐linked ubiquitin chains, thereby increasing STAT3 protein stability and regulating microglial inflammatory responses. These findings not only advance our understanding of the mechanistic role of deubiquitinating enzymes in microglial function but also provide experimental evidence supporting OTUD7B as a potential therapeutic target for regulating STAT3 activity and intervening in neuroinflammation and Alzheimer's disease.

## Author Contributions

L.L., H.T., and L.X. designed and performed the majority of the experiments and analyzed the data. Q.Y., Y.Z., Y.K., B.S., J.S., and C.H. provided technical support, essential reagents, and assisted with data collection. L.S., A.V.S., Z.W., and H.C. supervised parts of the research. L.L. wrote the initial manuscript draft. Y.W., X.Z., and G.C. conceived and supervised the entire project, secured funding, and reviewed and edited the manuscript. All authors discussed the results and commented on the manuscript.

## Conflicts of Interest

The authors declare no conflicts of interest.

## Supporting information




**Supporting File 1**: advs77235‐sup‐0001‐SuppMat.docx.

**Supporting File 2**: advs77235‐sup‐0002‐Data.zip.

## Data Availability

The data that support the findings of this study are available in the supplementary material of this article.

## References

[1] P. Scheltens , B. De Strooper , M. Kivipelto , et al., “Alzheimer's disease,” The Lancet 397, no. 10284 (2021): 1577–1590, 10.1016/S0140-6736(20)32205-4. PMC8354300 33667416

[2] L. Lannfelt , N. R. Relkin , and E. R. Siemers , “Amyloid‐ß‐Directed Immunotherapy for Alzheimer's Disease,” Journal of Internal Medicine 275, no. 3 (2014): 284–295, 10.1111/joim.12168. 24605809 PMC4238820

[3] S. A. Small and K. Duff , “Linking Aβ and Tau in Late‐Onset Alzheimer's Disease: A Dual Pathway Hypothesis,” Neuron 60, no. 4 (2008): 534–542, 10.1016/j.neuron.2008.11.007. 19038212 PMC2692134

[4] J. M. Long and D. M. Holtzman , “Alzheimer Disease: An Update on Pathobiology and Treatment Strategies,” Cell 179, no. 2 (2019): 312–339, 10.1016/j.cell.2019.09.001. 31564456 PMC6778042

[5] Y. Su , H. Li , W. Zhang , et al., “Connexin43 hemichannel Blockade Turns Microglia Neuroprotective and Mitigates Cognitive Deficits in a Mouse Model of Amyloidosis,” Nature Communications 16, no. 1 (2025): 5621, 10.1038/s41467-025-60746-w. PMC12219535 40595567

[6] M. T. Heneka , W. M. van der Flier , F. Jessen , et al., “Neuroinflammation in Alzheimer Disease,” Nature Reviews Immunology 25, no. 5 (2025): 321–352, 10.1038/s41577-024-01104-7. PMC13148177 39653749

[7] J. Schwartzentruber , S. Cooper , J. Z. Liu , et al., “Genome‐Wide Meta‐Analysis, Fine‐mapping and Integrative Prioritization Implicate New Alzheimer's Disease Risk Genes,” Nature Genetics 53, no. 3 (2021): 392–402, 10.1038/s41588-020-00776-w. 33589840 PMC7610386

[8] A. A. Pimenova , T. Raj , and A. M. Goate , “Untangling Genetic Risk for Alzheimer's Disease,” Biological Psychiatry 83, no. 4 (2018): 300–310, 10.1016/j.biopsych.2017.05.014. 28666525 PMC5699970

[9] G. Novikova , M. Kapoor , J. TCW , et al., “Integration of Alzheimer's Disease Genetics and Myeloid Genomics Identifies Disease Risk Regulatory Elements and Genes,” Nature Communications 12, no. 1 (2021): 1610, 10.1038/s41467-021-21823-y. PMC7955030 33712570

[10] H. Wake , A. J. Moorhouse , and J. Nabekura , “Functions of Microglia in the Central Nervous System—Beyond the Immune Response,” Neuron Glia Biology 7, no. 1 (2011): 47–53, 10.1017/S1740925X12000063. 22613055

[11] M. Prinz , T. Masuda , M. A. Wheeler , and F. J. Quintana , “Microglia and Central Nervous System–Associated Macrophages—From Origin to Disease Modulation,” Annual Review of Immunology 39, no. 1 (2021): 251–277, 10.1146/annurev-immunol-093019-110159. PMC8085109 33556248

[12] K. N. Swatek and D. Komander , “Ubiquitin Modifications,” Cell Research 26, no. 4 (2016): 399–422, 10.1038/cr.2016.39. 27012465 PMC4822133

[13] O. Braten , I. Livneh , T. Ziv , et al., “Numerous Proteins With Unique Characteristics are Degraded by the 26S Proteasome Following Monoubiquitination,” Proceedings of the National Academy of Sciences 113, no. 32 (2016): E4639–E4647, 10.1073/pnas.1608644113. PMC4987823 27385826

[14] B. Liu , J. Ruan , M. Chen , et al., “Deubiquitinating Enzymes (DUBs): Decipher Underlying Basis of Neurodegenerative Diseases,” Molecular Psychiatry 27, no. 1 (2022): 259–268, 10.1038/s41380-021-01233-8. 34285347

[15] T. E. T. Mevissen , M. K. Hospenthal , P. P. Geurink , et al., “OTU Deubiquitinases Reveal Mechanisms of Linkage Specificity and Enable Ubiquitin Chain Restriction Analysis,” Cell 154, no. 1 (2013): 169–184, 10.1016/j.cell.2013.05.046. 23827681 PMC3705208

[16] Y. Deng and P. Liu , “OTU Deubiquitinases in Disease: Roles and Targeting,” Trends in Molecular Medicine 32, no. 1 (2026): 84–97, 10.1016/j.molmed.2025.05.006. 40527635 PMC12354000

[17] R. Kumari , R. Kumar , S. Kumar , et al., “Amyloid Aggregates of the Deubiquitinase OTUB1 are Neurotoxic, Suggesting That They Contribute to the Development of Parkinson's Disease,” Journal of Biological Chemistry 295, no. 11 (2020): 3466–3484, 10.1074/jbc.RA119.009546. 32005664 PMC7076217

[18] Q. Xu , L. He , S. Zhang , X. Di , and H. Jiang , “Deubiquitinase OTUD3: A Double‐Edged Sword in Immunity and Disease,” Frontiers in Cell and Developmental Biology 11 (2023): 1237530, 10.3389/fcell.2023.1237530. 37829187 PMC10566363

[19] M. Qiu , W. Zhang , J. Dai , et al., “A20 Negatively Regulates Necroptosis‐Induced Microglia/Macrophages Polarization and Mediates Cerebral Ischemic Tolerance via Inhibiting the Ubiquitination of RIP3,” Cell death & disease 15, no. 12 (2024): 904, 10.1038/s41419-024-07293-2. 39695113 PMC11655947

[20] B. Wang , Z. Jie , D. Joo , et al., “TRAF2 and OTUD7B Govern a Ubiquitin‐Dependent Switch That Regulates mTORC2 Signalling,” Nature 545, no. 7654 (2017): 365–369, 10.1038/nature22344. 28489822 PMC5695540

[21] W. Xie , S. Tian , J. Yang , et al., “OTUD7B deubiquitinates SQSTM1/p62 and Promotes IRF3 Degradation to Regulate Antiviral Immunity,” Autophagy 18, no. 10 (2022): 2288–2302, 10.1080/15548627.2022.2026098. 35100065 PMC9542415

[22] Z. Gong , A. Li , J. Ding , et al., “OTUD7B Deubiquitinates LSD1 to Govern its Binding Partner Specificity, Homeostasis, and Breast Cancer Metastasis,” Advanced Science (Weinh) 8, no. 15 (2021): 2004504. 10.1002/advs.202004504 PMC8336515 34050636

[23] X. Zhao , J. Sun , L. Xiong , et al., “β‐amyloid Binds to Microglia Dectin‐1 to Induce Inflammatory Response in the Pathogenesis of Alzheimer's Disease,” International Journal of Biological Sciences 19, no. 10 (2023): 3249–3265, 10.7150/ijbs.81900. 37416769 PMC10321287

[24] W. Ali , M. Ikram , H. Y. Park , et al., “Oral Administration of Alpha Linoleic Acid Rescues Aβ‐Induced Glia‐Mediated Neuroinflammation and Cognitive Dysfunction in C57BL/6N Mice,” Cells 9, no. 3 (2020): 667, 10.3390/cells9030667. 32182943 PMC7140708

[25] X.‐D. Pan , Y.‐G. Zhu , N. Lin , et al., “Microglial Phagocytosis Induced by fibrillar β‐amyloid is Attenuated by oligomeric β‐amyloid: Implications for Alzheimer's Disease,” Molecular Neurodegeneration 6, no. 1 (2011): 45, 10.1186/1750-1326-6-45. 21718498 PMC3149591

[26] R.‐X. Dou , Y. Zhang , X.‐J. Hu , et al., “Aβ1‐42 Promotes Microglial Activation and Apoptosis in the Progression of AD by Binding to TLR4,” Redox Biology 78 (2024): 103428, 10.1016/j.redox.2024.103428. 39550828 PMC11615585

[27] Y. Zhao , X. He , X. Yang , et al., “CircFndc3b Mediates Exercise‐Induced Neuroprotection by Mitigating Microglial/Macrophage Pyroptosis via the ENO1/KLF2 Axis in Stroke Mice,” Advanced Science 12, no. 1 (2025): 2403818, 10.1002/advs.202403818. 39467260 PMC11714177

[28] M. Wittekindt , H. Kaddatz , S. Joost , et al., “Different Methods for Evaluating Microglial Activation Using Anti‐Ionized Calcium‐Binding Adaptor Protein‐1 Immunohistochemistry in the Cuprizone Model,” Cells 11, no. 11 (2022): 1723, 10.3390/cells11111723. 35681418 PMC9179561

[29] J. A. Harrigan , X. Jacq , N. M. Martin , and S. P. Jackson , “Deubiquitylating Enzymes and Drug Discovery: Emerging Opportunities,” Nature Reviews Drug Discovery 17, no. 1 (2018): 57–78, 10.1038/nrd.2017.152. 28959952 PMC7097658

[30] Z. Wei , K. Zeng , J. Hu , et al., “USP10 deubiquitinates Tau, Mediating its Aggregation,” Cell death & disease 13, no. 8 (2022): 726, 10.1038/s41419-022-05170-4. 35987808 PMC9392799

[31] S. Kolla , M. Ye , and K. G. Mark , “Assembly and Function of Branched Ubiquitin Chains,” Trends in Biochemical Sciences 47, no. 9 (2022): 759–771, 10.1016/j.tibs.2022.04.003. 35508449

[32] C. Niemand , A. Nimmesgern , S. Haan , et al., “Activation of STAT3 by IL‐6 and IL‐10 in Primary Human Macrophages is Differentially Modulated by Suppressor of Cytokine Signaling 3,” The Journal of Immunology 170, no. 6 (1950): 3263–3272, 10.4049/jimmunol.170.6.3263. 12626585

[33] Z.‐H. Wang , J. Xiang , X. Liu , et al., “Deficiency in BDNF/TrkB Neurotrophic Activity Stimulates δ‐Secretase by Upregulating C/EBPβ in Alzheimer's Disease,” Cell Reports 28, no. 3 (2019): 655–669.e5, 10.1016/j.celrep.2019.06.054. 31315045 PMC6684282

[34] L. Liu , K. M. McBride , and N. C. Reich , “STAT3 Nuclear Import is Independent of Tyrosine Phosphorylation and Mediated by importin‐α3,” Proceedings of the National Academy of Sciences 102, no. 23 (2005): 8150–8155, 10.1073/pnas.0501643102. PMC1149424 15919823

[35] N. C. Reich and L. Liu , “Tracking STAT Nuclear Traffic,” Nature reviews Immunology 6, no. 8 (2006): 602–612, 10.1038/nri1885. 16868551

[36] H. Y. Nam , J. H. Nam , G. Yoon , et al., “Ibrutinib Suppresses LPS‐Induced Neuroinflammatory Responses in BV2 Microglial Cells and Wild‐Type Mice,” Journal of Neuroinflammation 15, no. 1 (2018): 271, 10.1186/s12974-018-1308-0. 30231870 PMC6145206

[37] J. Mehla , I. Singh , D. Diwan , et al., “STAT3 Inhibitor Mitigates Cerebral Amyloid Angiopathy and Parenchymal Amyloid Plaques While Improving Cognitive Functions and Brain Networks,” Acta Neuropathologica Communications 9, no. 1 (2021): 193, 10.1186/s40478-021-01293-5. 34911575 PMC8672532

[38] S. Yu , L. S. Gong , N. F. Li , Y. F. Pan , and Z. L. Galangin , “Galangin (GG) Combined With cisplatin (DDP) to Suppress Human Lung Cancer by Inhibition of STAT3‐Regulated NF‐κB and Bcl‐2/Bax Signaling Pathways,” Biomedicine & Pharmacotherapy 97 (2018): 213–224, 10.1016/j.biopha.2017.10.059. 29091869

[39] B. Ye , W. Lin , Y. Jiang , et al., “Cardiomyocyte‐Derived YOD1 Promotes Pathological Cardiac Hypertrophy by Deubiquitinating and Stabilizing STAT3,” Science Advances 11, no. 26 (2025): adu8422, 10.1126/sciadv.adu8422. PMC12189954 40561034

[40] C. Zenge and A. Ordureau , “Ubiquitin System Mutations in Neurological Diseases,” Trends in Biochemical Sciences 49, no. 10 (2024): 875–887, 10.1016/j.tibs.2024.06.011. 38972780 PMC11455613

[41] D. M. Samhadaneh , K. A. Alqarni , A. Smart , et al., “Gold Nanourchins Induce Cellular Stress, Impair Proteostasis and Damage RNA,” Nanomedicine: Nanotechnology, Biology and Medicine 22 (2019): 102083, 10.1016/j.nano.2019.102083. 31404650

[42] D. Komander , M. J. Clague , and S. Urbe , “Breaking the Chains: Structure and Function of the Deubiquitinases,” Nature Reviews Molecular Cell Biology 10, no. 8 (2009): 550–563, 10.1038/nrm2731. 19626045

[43] N. J. Schauer , R. S. Magin , X. Liu , L. M. Doherty , and S. J. Buhrlage , “Advances in Discovering Deubiquitinating Enzyme (DUB) Inhibitors,” Journal of Medicinal Chemistry 63, no. 6 (2020): 2731–2750, 10.1021/acs.jmedchem.9b01138. 31682427

[44] Z. Zhang , S. Chen , S. Jun , et al., “MLKL‐USP7‐UBA52 Signaling is Indispensable for Autophagy in Brain Through Maintaining Ubiquitin Homeostasis,” Autophagy 21, no. 2 (2025): 424–446, 10.1080/15548627.2024.2395727. 39193909 PMC11759533

[45] Y. Guo , C. Cai , B. Zhang , et al., “Targeting USP11 Regulation by a Novel Lithium‐Organic Coordination Compound Improves Neuropathologies and Cognitive Functions in Alzheimer Transgenic Mice,” EMBO Molecular Medicine 16, no. 11 (2024): 2856–2881, 10.1038/s44321-024-00146-7. 39394468 PMC11555261

[46] L. Ding , L. Lu , S. Zheng , et al., “Usp14 Deficiency Removes α‐synuclein by Regulating S100A8/A9 in Parkinson's Disease,” Cellular and Molecular Life Sciences 81, no. 1 (2024): 232, 10.1007/s00018-024-05246-8. 38780644 PMC11116365

[47] A. Siwach , H. Patel , A. Khairnar , and P. Parekh , “Molecular Symphony of Mitophagy: Ubiquitin‐Specific Protease‐30 as a Maestro for Precision Management of Neurodegenerative Diseases,” CNS Neuroscience & Therapeutics 31, no. 1 (2025): 70192, 10.1111/cns.70192. PMC11751875 39840724

[48] Y. Sui , Z. Shen , X. Li , et al., “Rupatadine‐Inhibited OTUD3 Promotes DLBCL Progression and Immune Evasion Through Deubiquitinating MYL12A and PD‐L1,” Cell death & disease 15, no. 8 (2024): 561, 10.1038/s41419-024-06941-x. 39097608 PMC11297949

[49] X. Song , T. Liu , L. Yu , et al., “OTUD5 Protects Dopaminergic Neurons by Promoting the Degradation of α‐Synuclein in Parkinson's Disease Model,” Advanced Science 12, no. 7 (2025): 2406700, 10.1002/advs.202406700. 39721018 PMC11831440

[50] M. F. Gulen , N. Samson , A. Keller , et al., “cGAS–STING Drives Ageing‐Related Inflammation and Neurodegeneration,” Nature 620, no. 7973 (2023): 374–380, 10.1038/s41586-023-06373-1. 37532932 PMC10412454

[51] D. Wang , X. Gu , X. Ma , et al., “Nanopolyphenol Rejuvenates Microglial Surveillance of Multiple Misfolded Proteins Through Metabolic Reprogramming,” Acta Pharmaceutica Sinica B 13, no. 2 (2023): 834–851, 10.1016/j.apsb.2022.07.014. 36873190 PMC9978858

[52] M. Tzioras , R. I. McGeachan , and C. S. Durrant , “Synaptic Degeneration in Alzheimer Disease,” Nature Reviews Neurology 19, no. 1 (2023): 19–38, 10.1038/s41582-022-00749-z. 36513730

[53] T. Arendt , “Synaptic Degeneration in Alzheimer's Disease,” Acta Neuropathologica 118, no. 1 (2009): 167–179, 10.1007/s00401-009-0536-x. 19390859

[54] S. Zhao , L. Wang , D. Kleidonas , et al., “Chemogenetic Activation of Microglial Gi Signaling Decreases Microglial Surveillance and Impairs Neuronal Synchronization,” Science Advances 11, no. 9 (2025): ado7829, 10.1126/sciadv.ado7829. PMC11870068 40020068

[55] T. Liu , L. Wang , P. Liang , et al., “USP19 Suppresses Inflammation and Promotes M2‐Like Macrophage Polarization by Manipulating NLRP3 Function via Autophagy,” Cellular & Molecular Immunology 18, no. 10 (2021): 2431–2442, 10.1038/s41423-020-00567-7. 33097834 PMC8484569

[56] L. She , L. Li , H. Tang , et al., “OTUD1 Positively Regulates Microglia Neuroinflammation and Promotes the Pathogenesis of Alzheimer's Disease by deubiquitinating C/EBPβ,” Acta Pharmacologica Sinica 46, no. 10 (2025): 2608–2621, 10.1038/s41401-025-01566-y. 40335710 PMC12460882

[57] J. Sun , F. Chen , L. She , et al., “YOD1 Regulates Microglial Homeostasis by deubiquitinating MYH9 to Promote the Pathogenesis of Alzheimer's Disease,” Acta Pharmaceutica Sinica B 15, no. 1 (2025): 331–348, 10.1016/j.apsb.2024.11.020. 40041897 PMC11873648

[58] K. Harit , W. Yi , A. Jeron , et al., “Astrocytic‐OTUD7B Ameliorates Murine Experimental Autoimmune Encephalomyelitis by Stabilizing Glial Fibrillary Acidic Protein and Preventing Inflammation,” Nature Communications 16, no. 1 (2025): 9279, 10.1038/s41467-025-65093-4. PMC12537900 41115891

[59] M. S. Ritorto , R. Ewan , A. B. Perez‐Oliva , et al., “Screening of DUB Activity and Specificity by MALDI‐TOF Mass Spectrometry,” Nature Communications 5, no. 1 (2014): 4763, 10.1038/ncomms5763. PMC4147353 25159004

[60] A. Martinez‐Ferriz , A. Ferrando , A. Fathinajafabadi , and R. Farras , “Ubiquitin‐Mediated Mechanisms of Translational Control,” Seminars in Cell & Developmental Biology 132 (2022): 146–154, 10.1016/j.semcdb.2021.12.009. 34952788

[61] S. Tian , S. Jin , Y. Wu , et al., “High‐Throughput Screening of Functional Deubiquitinating Enzymes in Autophagy,” Autophagy 17, no. 6 (2021): 1367–1378, 10.1080/15548627.2020.1761652. 32453962 PMC8205047

[62] A. C. Graham , E. Bellou , J. C. Harwood , et al., “Human Longevity and Alzheimer's Disease Variants act via Microglia and Oligodendrocyte Gene Networks,” Brain 148, no. 3 (2025): 969–984, 10.1093/brain/awae339. 39778705 PMC11884759

[63] J. A. Roberts , V. R. Varma , Y. An , et al., “A Brain Proteomic Signature of Incipient Alzheimer's Disease in Young APOE ε4 Carriers Identifies Novel Drug Targets,” Science Advances 7, no. 46 (2021): abi8178, 10.1126/sciadv.abi8178. PMC8580310 34757788

[64] E. Kim , H. Kim , M. P. Jedrychowski , et al., “Irisin Reduces amyloid‐β by Inducing the Release of neprilysin From Astrocytes Following Downregulation of ERK‐STAT3 Signaling,” Neuron 111, no. 22 (2023): 3619–3633.e8, 10.1016/j.neuron.2023.08.012. 37689059 PMC10840702

[65] X. Mi , X. Ruan , R. Lin , et al., “Intranasal Administration of Ganoderma Lucidum‐Derived Exosome‐Like Nanovesicles Ameliorates Cognitive Impairment by Reducing Inflammation in a Mouse Model of Alzheimer's Disease,” Frontiers in Pharmacology 16 (2025): 1572771, 10.3389/fphar.2025.1572771. 40689203 PMC12271231

[66] X. Chen , Y. Gan , K. Zhang , et al., “MicroRNA‐204‐5p Deficiency Within the vmPFC Region Contributes to Neuroinflammation and Behavioral Disorders via the JAK2/STAT3 Signaling Pathway in Rats,” Advanced Science 12, no. 10 (2025): 2403080, 10.1002/advs.202403080. 39792918 PMC11905084

[67] H. Wang , L. Chao , S. Shen , et al., “Exploring the Pharmacological Mechanism of Bu‐Wang San on Alzheimer's Disease Through Multiple GEO Datasets of the Human Hippocampus, Network Pharmacology, and Metabolomics Based on GC‐MS and UPLC‐Q/TOF‐MS,” Journal of Ethnopharmacology 350 (2025): 119994, 10.1016/j.jep.2025.119994. 40389089

[68] S. Zou , Q. Tong , B. Liu , W. Huang , Y. Tian , and X. Fu , “Targeting STAT3 in Cancer Immunotherapy,” Molecular Cancer 19, no. 1 (2020): 145, 10.1186/s12943-020-01258-7. 32972405 PMC7513516

[69] V. R. Varma , R. J. Desai , S. Navakkode , et al., “Hydroxychloroquine Lowers Alzheimer's Disease and Related Dementias Risk and Rescues Molecular Phenotypes Related to Alzheimer's Disease,” Molecular Psychiatry 28, no. 3 (2023): 1312–1326, 10.1038/s41380-022-01912-0. 36577843 PMC10005941

[70] G. Yang , Y. Ren , P. Zhong , et al., “Histone Demethylase PHF2 Regulates Inflammatory Genes in Alzheimer's Disease,” Molecular Psychiatry 31, no. 2 (2026): 845–859, 10.1038/s41380-025-03181-z. 40849543 PMC12746590

[71] A. Vuorinen , C. R. Kennedy , K. A. McPhie , et al., “Enantioselective OTUD7B Fragment Discovery Through Chemoproteomics Screening and High‐throughput Optimisation,” Communications Chemistry 8, no. 1 (2025): 12, 10.1038/s42004-025-01410-8. 39809917 PMC11732987

