# Supplementary material for: Microglial Deubiquitinase OTUD7B Stabilizes STAT3 to Promote Neuroinflammation and Cognitive Decline in Alzheimer's Disease
Source: Adv Sci (Weinh). 2026 Aug 19:e23043. Online ahead of print. doi: 10.1002/advs.202523043 (PMC13487723; doi:10.1002/advs.202523043)
Supplement: Supplementary file 1 [file ADVS-9999-e23043-s002.docx]

*Supporting information*

**Microglial deubiquitinase OTUD7B stabilizes STAT3 to promote neuroinflammation and cognitive decline in Alzheimer’s disease**

This supplementary file contains 4 supplementary tables and 11 supplementary figures.

*Supplementary tables*

**Supplementary Table S1. Sources of AAV9 Vectors**

| **Plasmid No** | **Vector information** |
| --- | --- |
| PT-3048 | rAAV-hSyn-DIO-mCherry -5'miR30-shRNA(scramble)-3'miR30-WPREs |
| PT-11867 | rAAV-CX3CR1-DIO-mCherry-5'miR30-shRNA1(OTUD7B)-3'miR30-WPREs |
| PT-0606 | rAAV-CX3CR1-CRE-WPRE-hGH pA |

**Supplementary Table S2. Sequences of siRNAs used in this study**

| **Gene** | **Species** | **Sequence (5’-3’)** |
| --- | --- | --- |
| *Otud7b* | mouse | GGCUCGAGAUCUUCUAGAATT  UUCUAGAAGAUCUCGAGCCTT |
| *OTUD7B* | human | GGCUGGUAUACACAGAAGATT  UCUUCUGUGUAUACCAGCCTT |
| *Stat3* | mouse | GGUCUCGGAAAUUUAACAUTT AUGUUAAAUUUCCGAGACCTT |

**Supplementary Table S3. Primer sequences for RT qPCR**

| **Gene** | **Species** | **Sequence (5’-3’)** |
| --- | --- | --- |
| *Tnf* | mouse | GGTGCCTATGTCTCAGCCTCTT  GCCATAGAACTGATGAGAGGGAG |
| *Il1b* | mouse | GCAACTGTTCCTGAACTCAACT  ATCTTTTGGGGTCCGTCAACT |
| *Il6* | mouse | CCTCTGGTCTTCTGGAGTA  ATGAATTGGATGGTCTTGGT |
| *Usp36* | mouse | CACCATCCATAGAACATCCA  CCTTCACATAGCAGTAGTAGT |
| *Tmem119* | mouse | ACTACCCATCCTCGTTCCCTGA  TAGCAGCCAGAATGTCAGCCTG |
| *Cd45* | mouse | CTTCAGTGGTCCCATTGTGGTG  TCAGACACCTCTGTCGCCTTAG |
| *Otud7b* | mouse | AGATGAGTGGCAGA  ATGAGCAAGGACGAAGAC AGGA |
| *Otud7b-KO* | mouse | GTAGCTCACAGTTTCAGCCTGGATATT  CCCAGCCTGTATGACTCTTTATATCTTC |
| *Gapdh* | mouse | TGCCCAGAACATCATCCCT  GGTCCTCAGTGTAGCCCAAG |

**Supplementary Table S4. Primer sequences for ChIP-qPCR**

| **Gene** | **Species** | **Sequence (5’-3’)** |
| --- | --- | --- |
| *TNF Promoter* | Homo | TTCAAGACCTTCTCCCCATTCTG GGTGACTTGATCAGGGAAGACTT |
| *IL1B Promoter* | Homo | GTAGAGTTTGCTGCTTGAATGGG CAAATACTTGCACAGAGGCTCAC |
| *IL6 Promoter* | Homo | TTTCCCAGGCTAGGATAACAGAG  AGGGAAAAGTGTCTCCTTCCTC |

*Supplementary figures*

**Figure S1.** **mRNA levels of** **OTUD7B are elevated in AD patients. (A-C)** mRNA levels of *Usp36* in the brain tissues of control and AD patients in GSE236562(A), GSE5281(B), and GSE173955(C). **(D-F)** mRNA levels of *Otud7b* in the brain tissues of control and AD patients inGSE236562(D), GSE5281(E), and GSE173955(F). **(G-I)** The percentages of OTUD7B^+^ cells in Figure 1I (G), Figure 1J (H), and Figure 1K (I) are shown (n=3). Statistical significance for all panels was determined by the unpaired Student’s *t*-test. Statistical data are presented as mean ± SEM.

**Figure S2.** **The deficiency of OTUD7B does not affect the survival of SH-SY5Y cells. (A)** The mean fluorescence intensity of OTUD7B in Figure 2E is presented. **(B)** SH-SY5Y cells were exposed to 20 μM of Aβ for 0, 3, 6, 12, and 24 hours, respectively. The protein levels of OTUD7B were measured by Western blotting. **(C)** Densitometric quantification for panel B. GAPDH serving as a loading control. **(D)** SH-SY5Y cells were exposed to different concentrations of Aβ for 24 hours. The protein levels of OTUD7B were measured by Western blotting. **(E)** Densitometric quantification for panel D. GAPDH serving as a loading control. **(F-G)** SH-SY5Y cells were transfected with si-OTUD7B. Control cells were transfected with negative control siRNA. Western blotting was used to determine OTUD7B protein levels (F), and densitometric quantification is shown (G). **(H-J)** SH-SY5Y cells were transfected with NC or si-OTUD7B. Cells were then challenged with Aβ (20 μM) for 24 hours. The mRNA levels of *Tnf* (H), *Il1b* (I), and *Il6* (J) were determined by RT-qPCR. **(K)** SH-SY5Y cells were treated as indicated in Panel H. The protein levels of BCL-2 and BAX in the cells were measured by Western blotting (J). GAPDH was used as the loading control. **(L)** Densitometric quantification of BCL-2/BAX levels in panel K is presented. **(M)** Apoptosis of SH-SY5Y cells was assessed by flow cytometry after treatment described in panel H. **(N)** Quantification of apoptotic SH-SY5Y cells in panel M. Statistical analysis was performed using non-linear regression analysis (C, E), unpaired Student’s *t*-test (G), and one-way ANOVA followed by Tukey’s multiple comparisons test (A, H-J, L, N). Statistical data are presented as mean ± SEM (n=3).

**Figure S3. OTUD7B deficiency reduces microglia-neuroinflammation and promotes neuronal survival. (A)** Schematic diagram of conditioned medium operation: BV2 cells were transfected with si-OTUD7B, while control cells were transfected with negative control siRNA. Then, both cell groups were treated with Aβ at 20 μM for 24 hours. The conditioned culture supernatant was collected to treat SH-SY5Y cells for 24 hours. The protein levels of BCL-2 and BAX were determined by Western blotting assay **(B).** GAPDH was used as the loading control. **(C)** Densitometric quantification of BCL-2/BAX levels in panel B is presented. **(D)** Apoptosis of SH-SY5Y cells was assessed by flow cytometry after treatment described in panel A. **(E)** Quantification of apoptotic SH-SY5Y cells in panel D. Statistical significance across all panels was determined by one-way ANOVA followed by Tukey's multiple-comparison test. Statistical data are presented as mean ± SEM (n=3).

**Figure S4. Validation of microglial OTUD7B knockdown.** Genetic identification of OTUD7B knockout mice. H_2_O served as the blank negative control, while WT refers to the wild-type mouse genotype. OTUD7B^CKO^ denotes the OTUD7B conditional knockout mouse genotype. **(A-B)** Immunofluorescence double staining confirmed that OTUD7B was down-regulated in microglia of 3×Tg mice injected with AAV-shOTUD7B (A) and qualification (B). Iba1: microglia marker, green; OTUD7B: red. **(C)**The PCR products for the OTUD7B^CKO^ mouse were 368 bp for Cx3cr1-Cre and 398 bp for Flox. **(D)** The histogram shows CD11b expression in pre-sorted cells (start.fcs), with 18.8% CD11b^+^ cells. **(E)** The histogram shows CD11b expression in post-sorted cells (iso.fcs), with 96.6% CD11b^+^ cells. **(F)** Quantitative analysis of CD11b^+^ cell percentages before and after sorting. **(G)** The protein levels of OTUD7B and Iba1 in the isolated microglia were measured by Western blotting. GAPDH was used as the loading control. **(H)** The densitometric quantification is shown. **(I)** Discrimination index (DI) in the novel object recognition test. **(J-K)** Immunofluorescence double staining confirmed that OTUD7B was down-regulated in microglia of OTUD7B^CKO^ mice (J) and qualification (K). **(L)** The protein levels of OTUD7B and Iba1 in the isolated microglia were measured by Western blotting. GAPDH was used as the loading control. **(M)** The densitometric quantification is shown. Statistical significance for all panels was determined by the unpaired Student’s *t*-test. Statistical data are presented as mean ± SEM (n=3).

**Figure S5. (A-B)** The mRNA levels of *Trem119* (A) and *Cd45* (B) in different groups of mice. Statistical significance was performed using one-way ANOVA followed by Tukey's multiple comparisons test (n=3). **(C-E)** The quantification of Aβ40 (C), Aβ42 (D) or Aβ40/ Aβ42 (E) in the hippocampus of 3×Tg mice using ELISA (n=6). Statistical significance across all panels was determined using one-way ANOVA followed by Tukey's multiple-comparison test. Statistical data are presented as mean ± SEM (n=3).

**Figure S6. STAT3 is a potential substrate of OTUD7B. (A)** A bubble plot was constructed, with the x-axis representing the enrichment ratio, the y-axis listing candidate proteins, and the size of each bubble indicating the corresponding MS signal intensity. **(B)** MS spectrum of the STAT3 peptide.

**Figure S7.** **OTUD7B regulated the protein stability of STAT3. (A-C)** BV2 cells were transfected with Flag or Flag-OTUD7B. Cells were then challenged with Aβ (20 μM) for 24 hours. Representative Western blotting images of p-STAT3 and STAT3 (A), and (B) the densitometric quantification is shown (n=3). Real-time qPCR analysis of mRNA levels of *Stat3* (C). **(D-E)** Representative Western blotting images of STAT3 in HEK293T cells transfected with Flag-OTUD7B plasmids, followed by a CHX pulse-chase assay (D), and the densitometric quantification is shown (E), n=3. **(F)** Densitometric quantification of STAT3/GAPDH levels in Figure 6F is presented, n=6. **(G)** Densitometric quantification of STAT3/GAPDH levels in Figure 6G is presented, n=6. **(H)** Representative Western blotting images of p-STAT3 and STAT3 in brain tissues of 3×Tg mice. **(I-J)** Densitometric quantification of p-STAT3/GAPDH (I) and STAT3/GAPDH (J) levels in panel H. Statistical analysis was performed using one-way ANOVA followed by Tukey’s multiple-comparison test (B, C, F, G) and unpaired Student’s *t*-test (E). Statistical data are presented as mean ± SEM.

**Figure S8.** **OTUD7B regulates STAT3 through the proteasome pathway. (A-B)** Immunoblot analysis of lysates from HEK293T cells transfected with Flag-OTUD7B. Cells were pretreated with DMSO (vehicle) or MG132 (10 μM) for 4 h before harvesting. (A) Representative Western blotting images of Flag and STAT3. (B) Densitometric quantification of STAT3/GAPDH levels in panel A is presented. **(C-D)** Immunoblot analysis of lysates from HEK293T cells transfected with Flag-OTUD7B. Cells were pretreated with DMSO (vehicle) or CQ (25 μM) for 4 h before harvesting. (C) Representative Western blotting images of Flag and STAT3. (D) Densitometric quantification of STAT3/GAPDH levels in panel C is presented. Statistical significance across all panels was determined using one-way ANOVA followed by Tukey's multiple-comparison test. Statistical data are presented as mean ± SEM (n=3).

**Figure S9.** **OTUD7B stabilizes STAT3 by removing K48-linked ubiquitin chains. (A)** Co-IP of STAT3 in HEK293T cells that were co-transfected with overexpression plasmids of His-STAT3, Flag-OTUD7B, Myc-Ub-K63, or Myc-Ub-K63R. **(B)** Co-IP of STAT3 in HEK293T cells that were co-transfected with overexpression plasmids encoding Flag-OTUD7B, His-STAT3, or Myc-Ub-K48. **(C-E)** BV2 cells were co-transfected with overexpression plasmids of Flag-OTUD7B, His-STAT3-WT, or His-STAT3-K283R. Cells were then challenged with Aβ (20 μM) for 24 hours. The mRNA levels of *Tnf* (C), *Il1b* (D), and *Il6* (E) were determined by RT-qPCR. Statistical significance across all panels was determined using one-way ANOVA followed by Tukey's multiple-comparison test. Statistical data are presented as mean ± SEM (n=3).

**Figure S10.** **OTUD7B deficiency suppresses the STAT3-NF-κB signaling pathway to alleviate neuroinflammation. (A)** BV2 cells were transfected with Flag or Flag-OTUD7B. Cells were then challenged with Aβ (20 μM) for 24 hours. Protein levels of STAT3 in nuclear and cytosolic fractions were determined by immunoblotting. Lamin B and GAPDH were used as loading controls. **(B)** Densitometric quantification of STAT3/Lamin B and STAT3/GAPDH levels in panel A is presented (n=3). **(C)** BV2 cells were treated as indicated in Panel A. STAT3 IP was performed, and levels of KPNA3 (importin-α3) were detected by immunoblotting. IgG IP was used as the negative control. **(D)** Densitometric quantification of KPNA3/STAT3 levels for panel C was presented (n=3). **(E)** The percentage of nuclear and cytoplasmic p65 shown in Figure 7E was quantitatively analyzed, and the data are presented as percentages. **(F-G)** BV2 cells were treated as indicated in Panel A. Representative images of NF-κB p65 (green) immunofluorescence staining in the BV2 cells (F) and the percentage of nuclear and cytoplasmic p65 (G). Scale bar = 20 µm. **(H)** Chromatin immunoprecipitation-qPCR analysis of STAT3 binding to candidate genes in HEK293T cells. RPL30 (ribosomal protein L30) was used as the positive control. Statistical significance was determined by two-way ANOVA followed by Tukey's multiple-comparison test (E, G), and others were determined by one-way ANOVA followed by Tukey's multiple-comparison test. Statistical data are presented as mean ± SEM (n=3).

**Figure S11. (A)** Quantification of the NeuN-positive area in Figure 8A is presented. **(B)** Quantification of the Iba1-positive area in Figure 8B is presented. Statistical significance was determined by two-way ANOVA followed by Tukey's multiple-comparison test. Statistical data are presented as mean ± SEM (n=3).
