# Supplementary material for: Microglial Deubiquitinase OTUD7B Stabilizes STAT3 to Promote Neuroinflammation and Cognitive Decline in Alzheimer's Disease
Source: Adv Sci (Weinh). 2026 Aug 19:e23043. Online ahead of print. doi: 10.1002/advs.202523043 (PMC13487723; doi:10.1002/advs.202523043)

Figure 1G

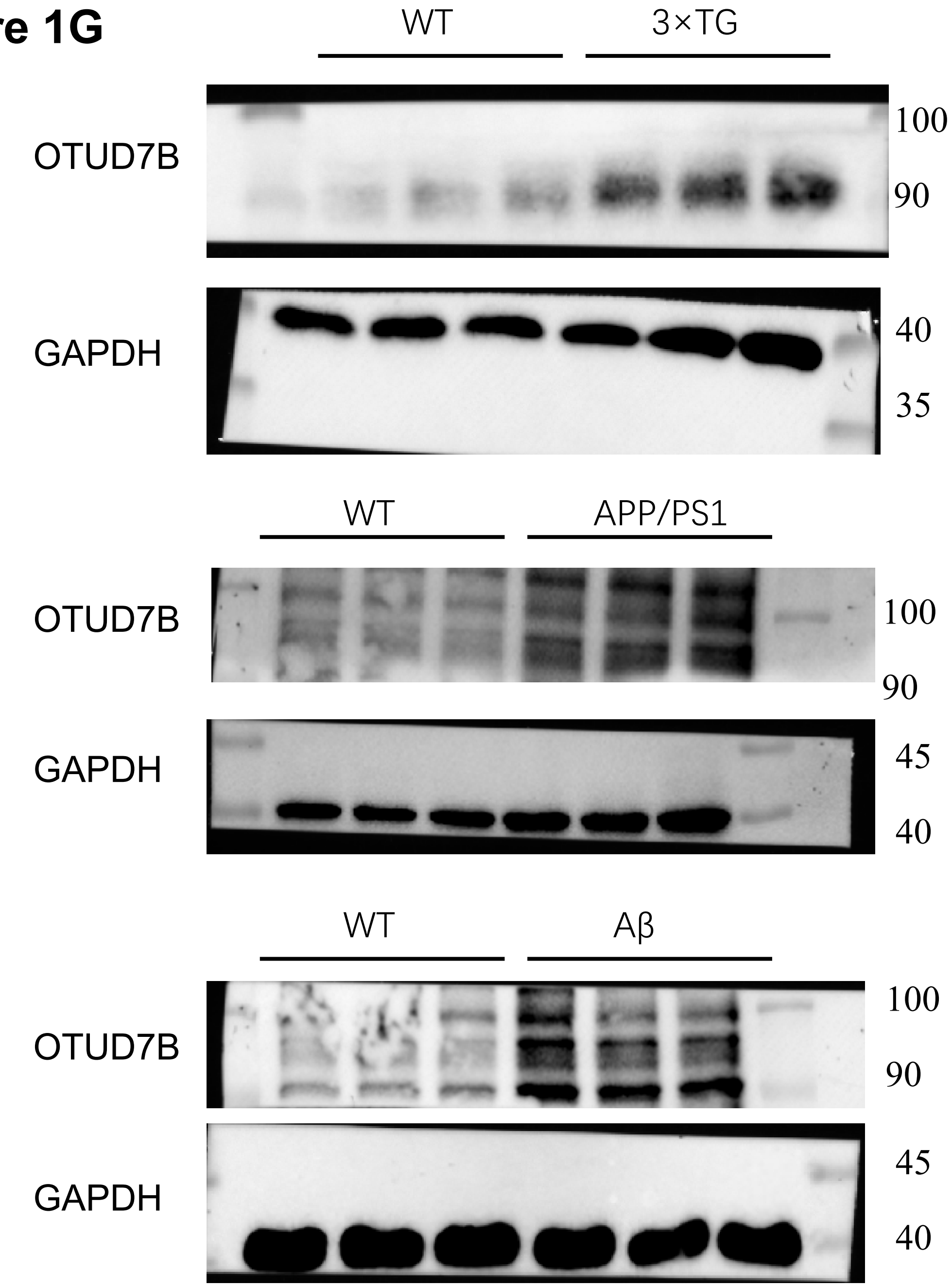

Figure 2A

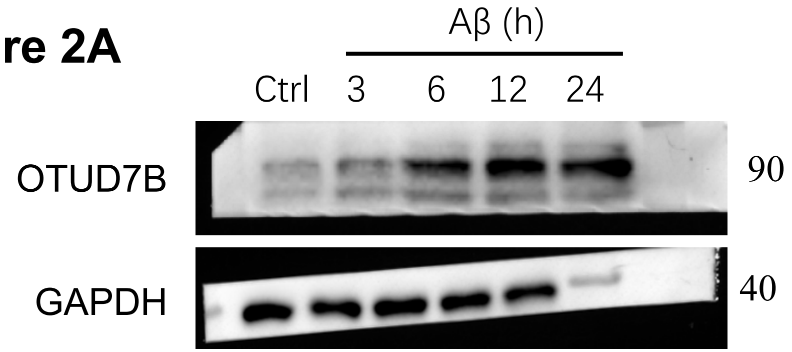

Figure 2C

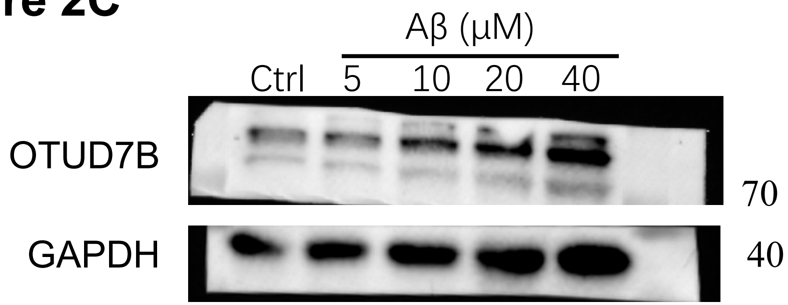

Figure 2F

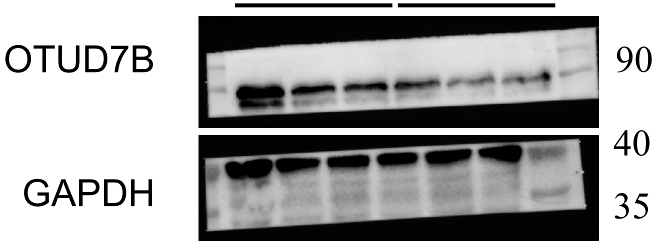

Figure 2K

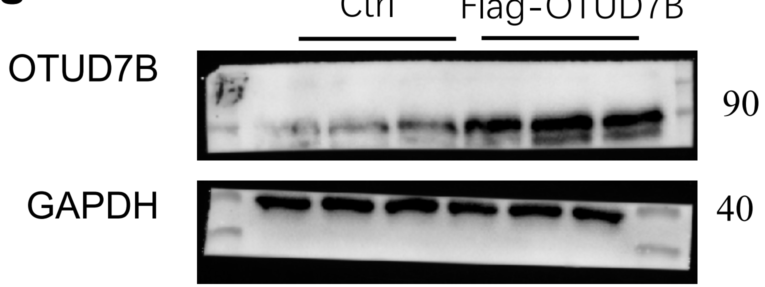

Figure S2A

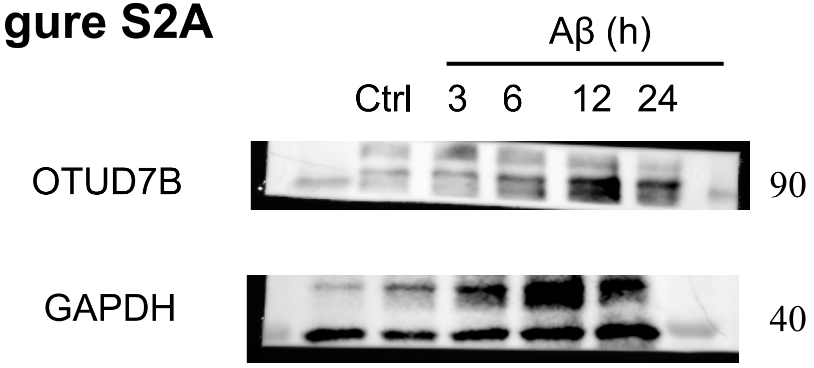

Figure S2D

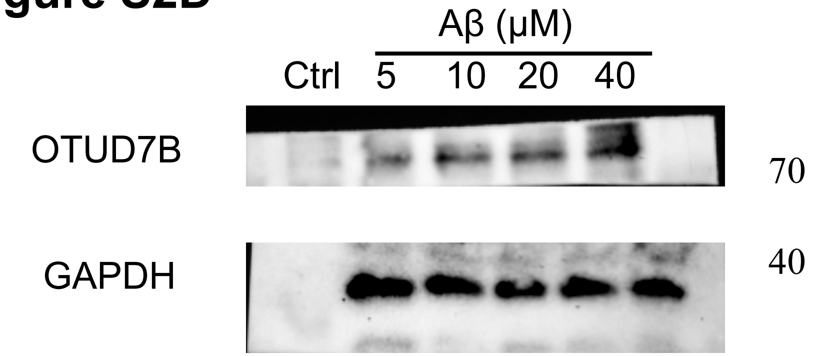

Figure S2F

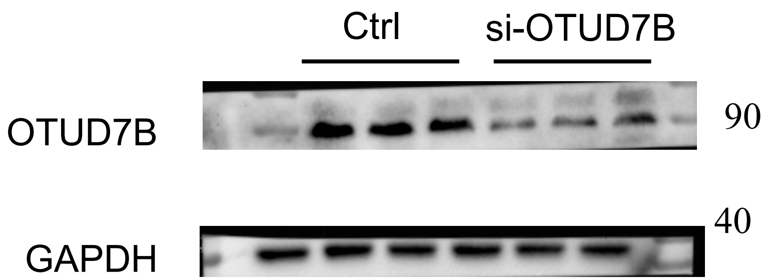

Figure S2K

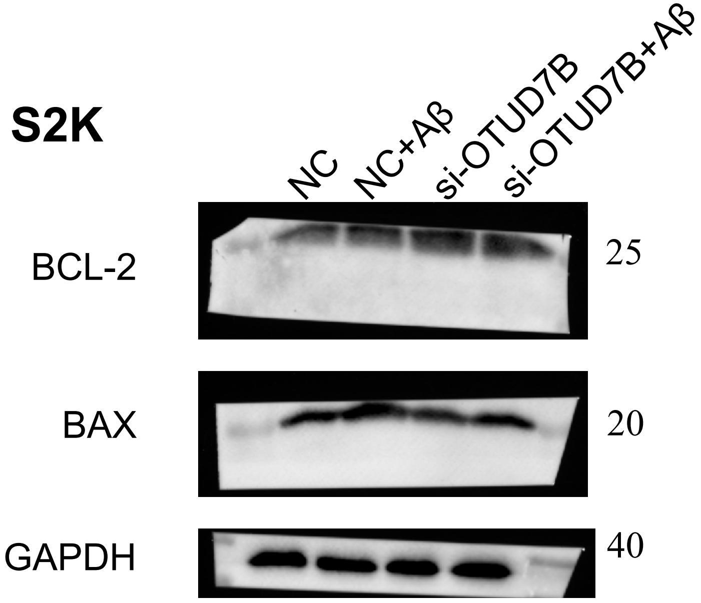

Figure 3P

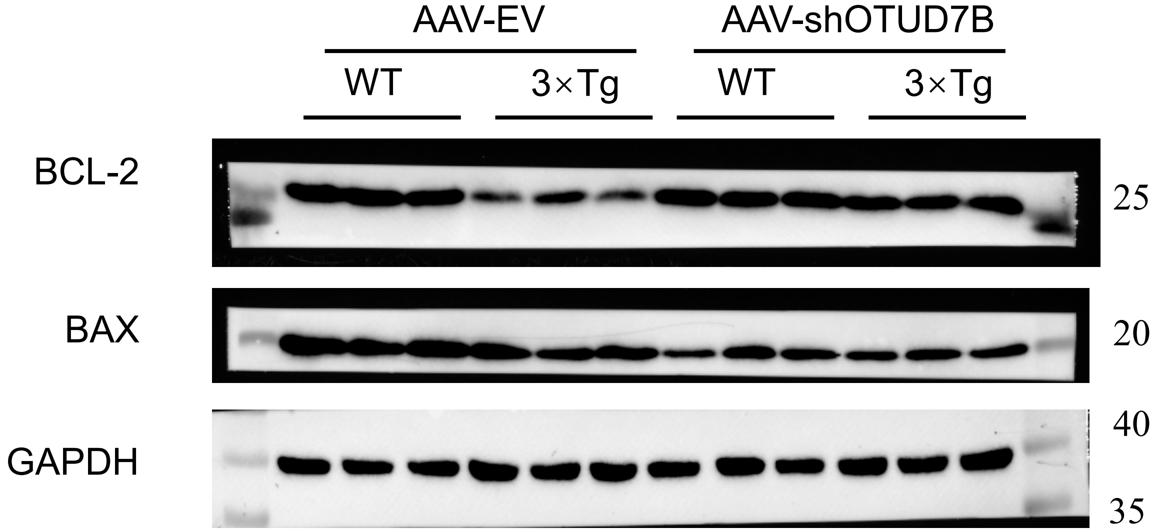

Figure S3B

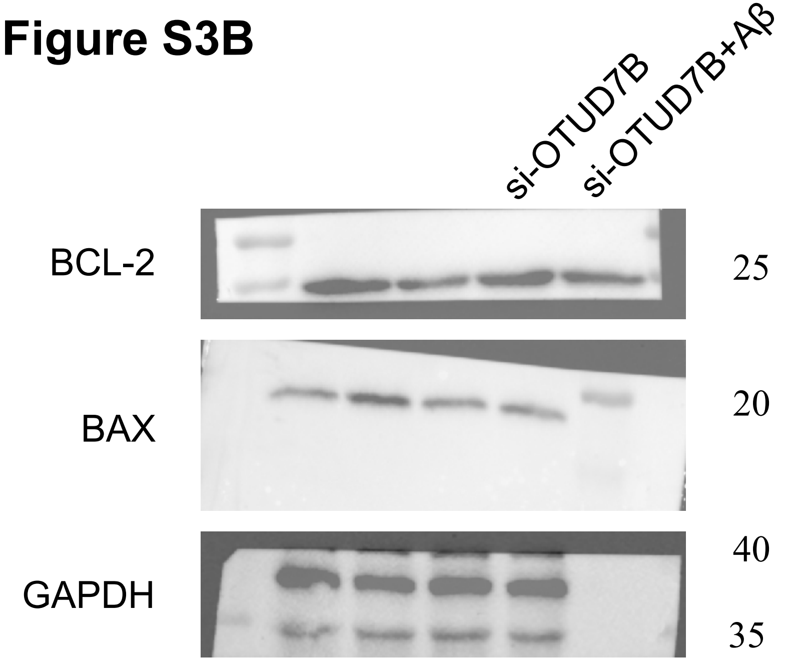

Figure 4P

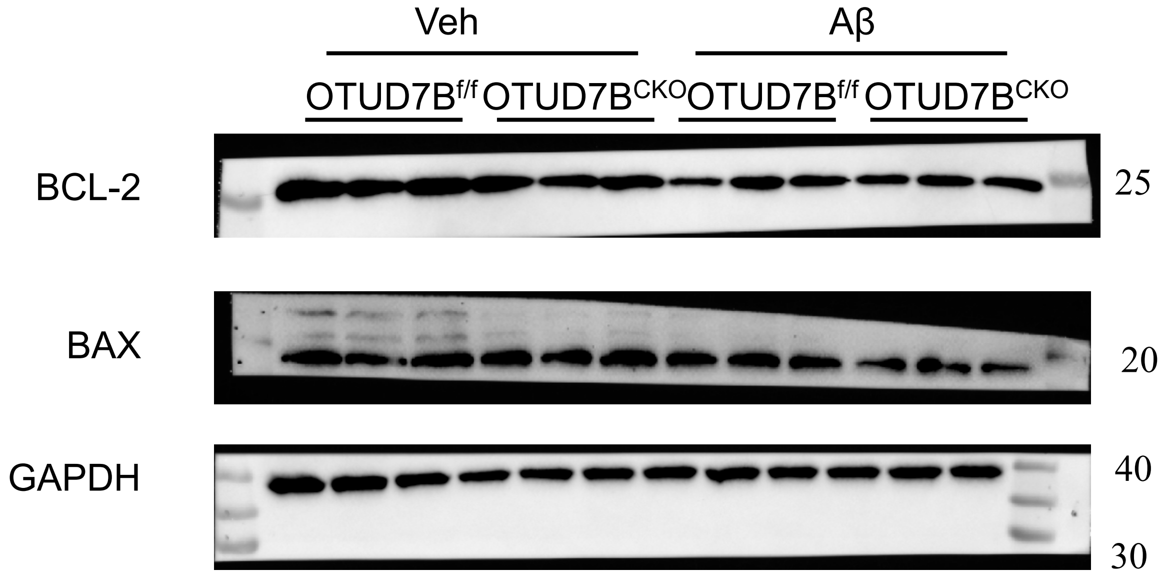

Figure S4E

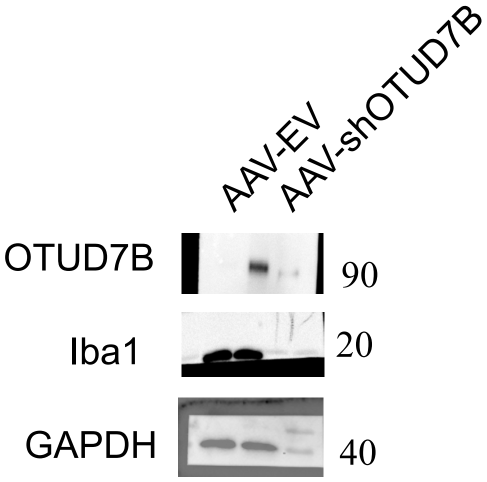

Figure S4H

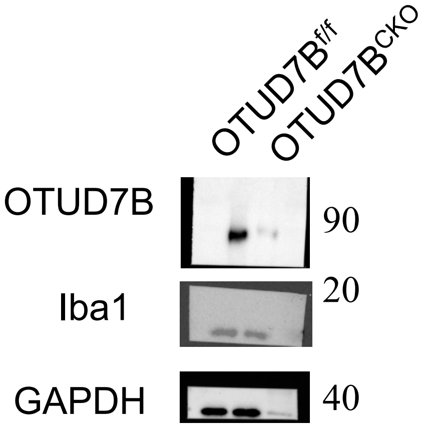

Figure 5B

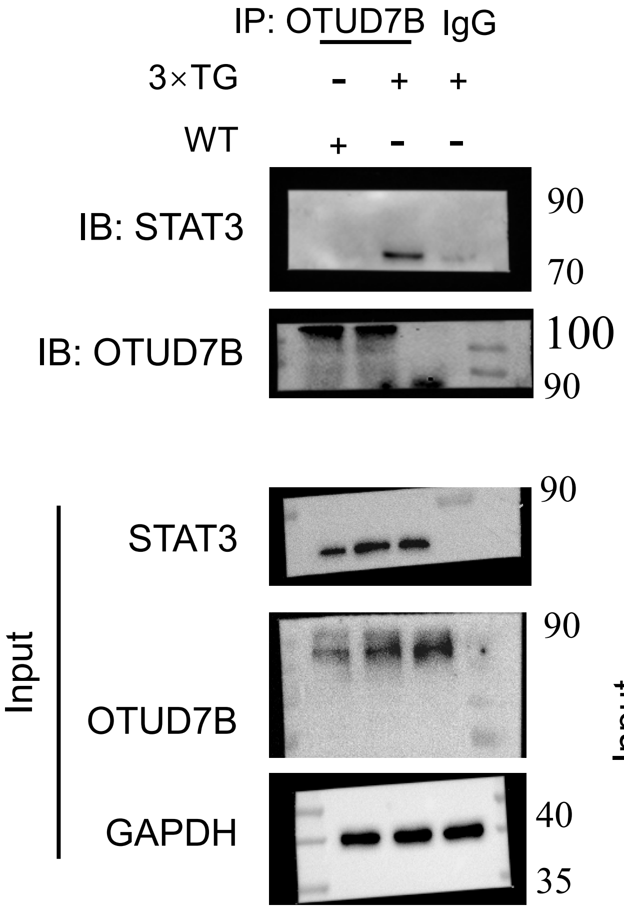

Figure 5C

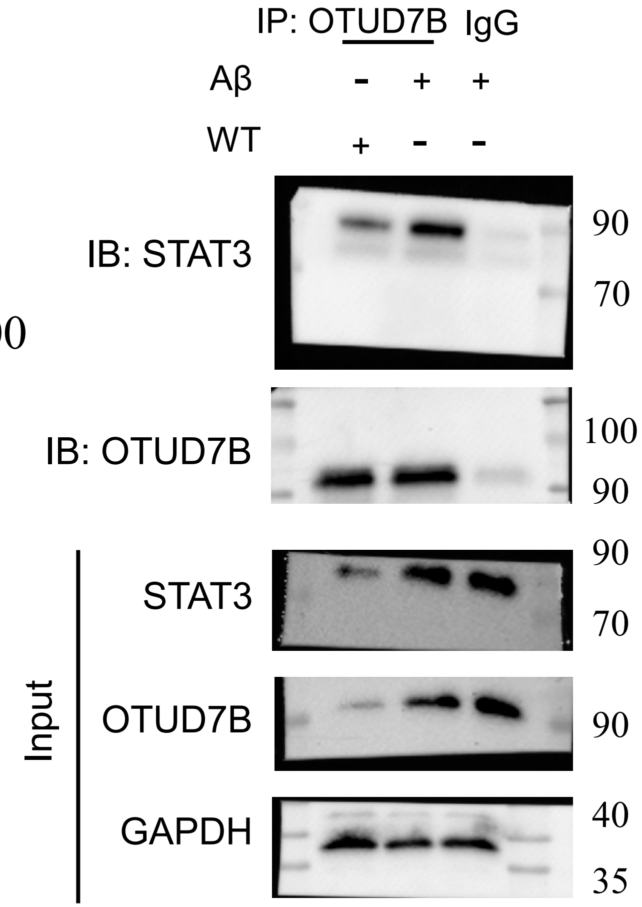

Figure 5D

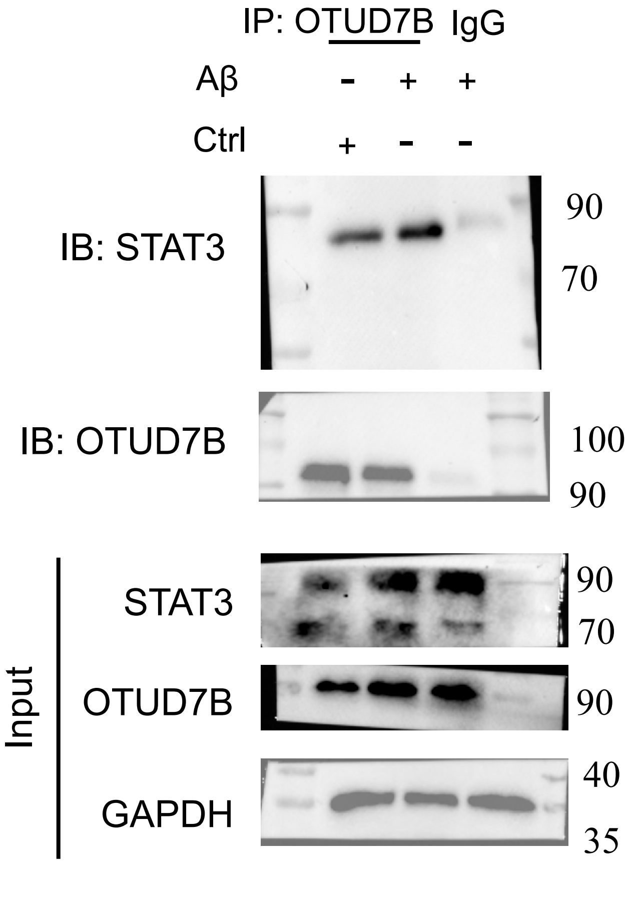

Figure 5H

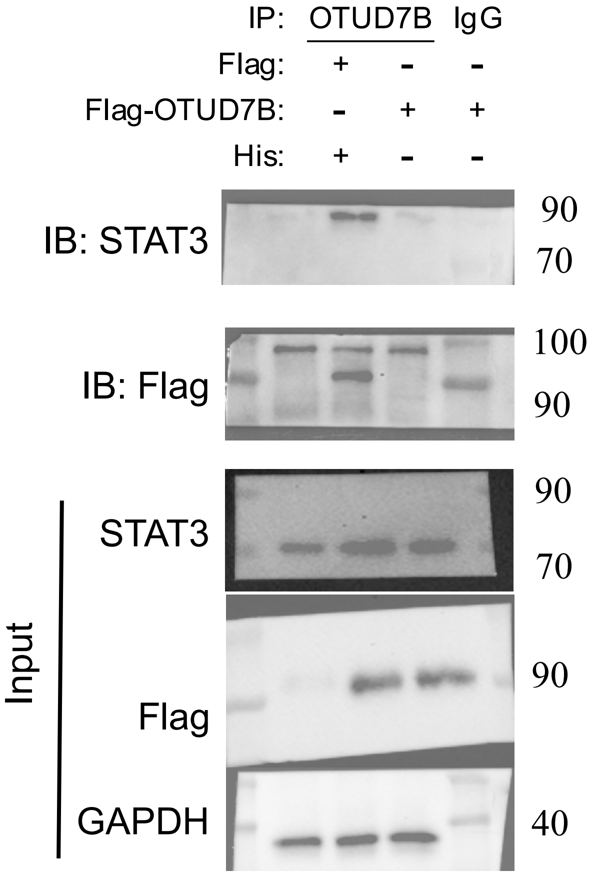

Figure 5J

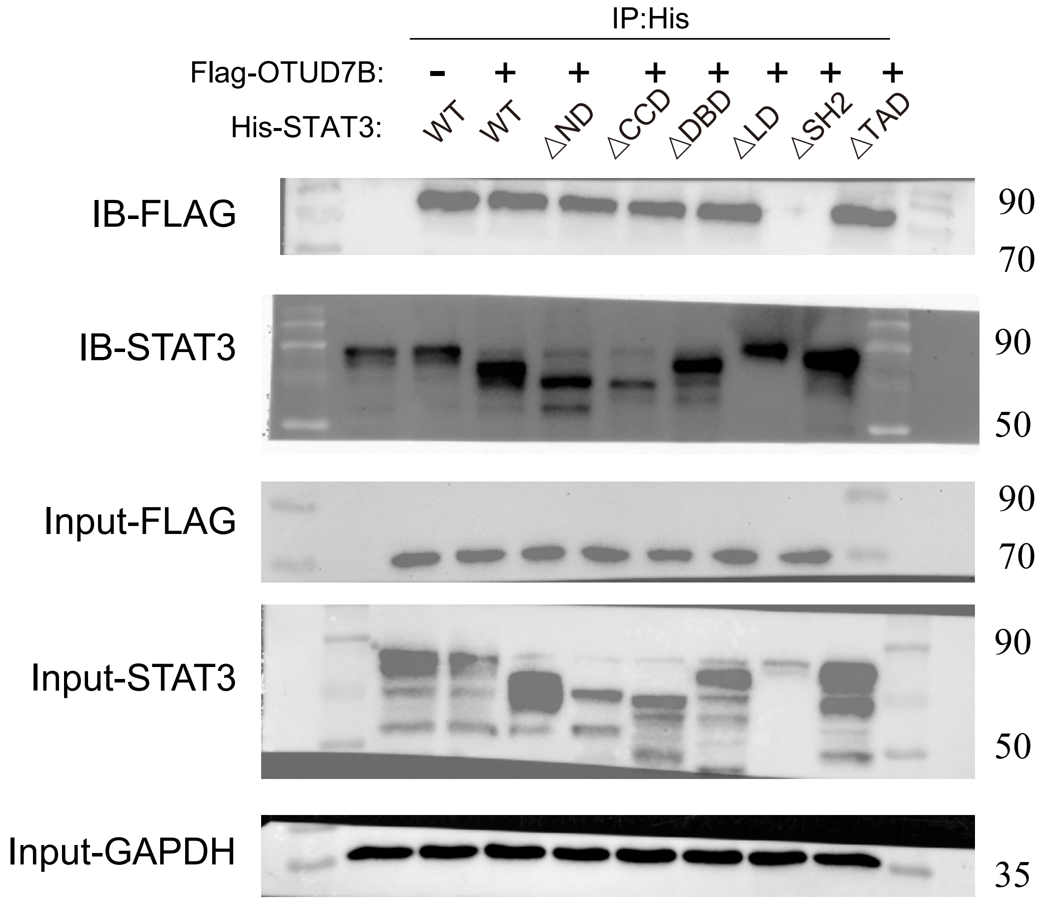

Figure 6A

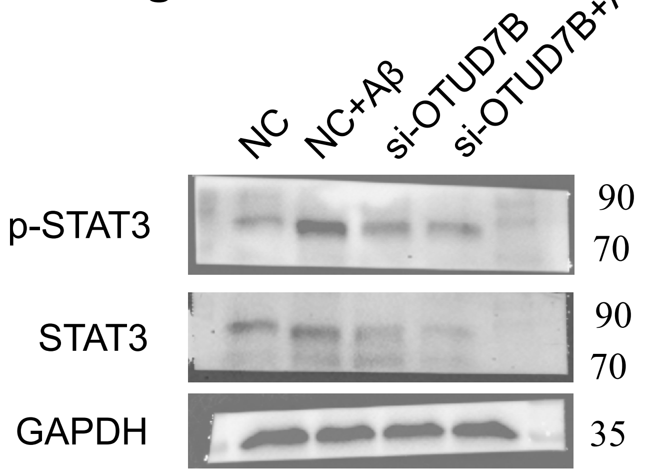

Figure 6D

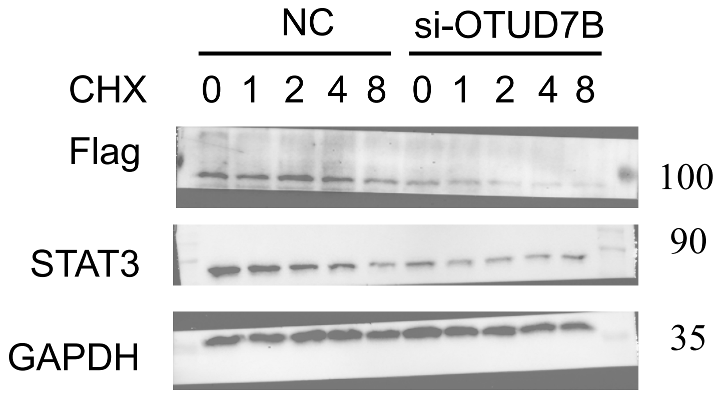

Figure 6F

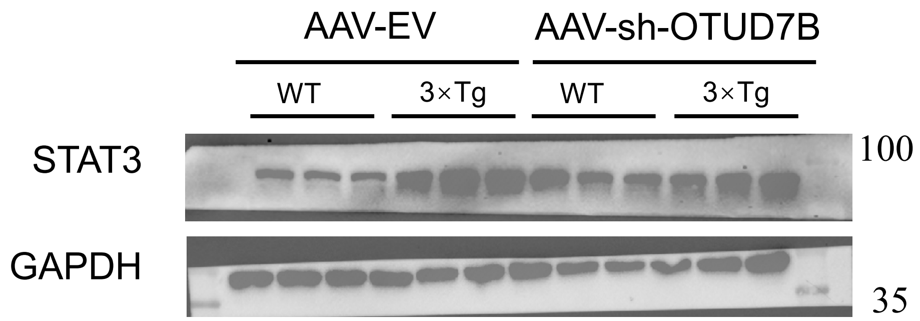

Figure 6G

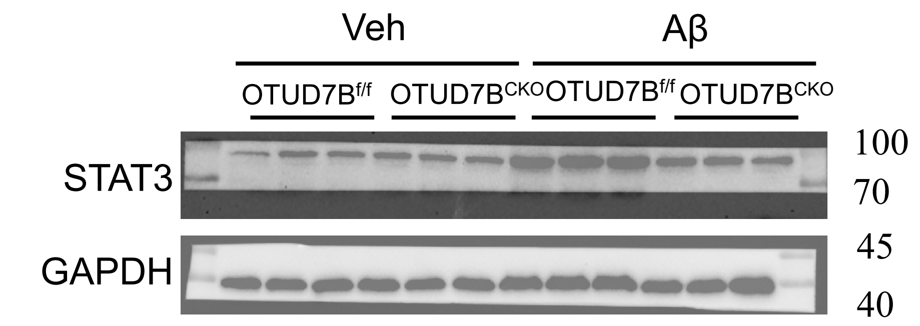

Figure S7A

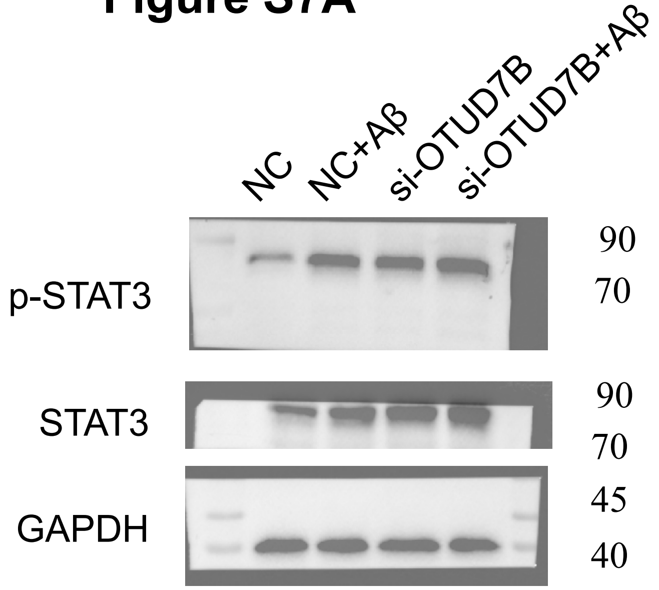

Figure S7D

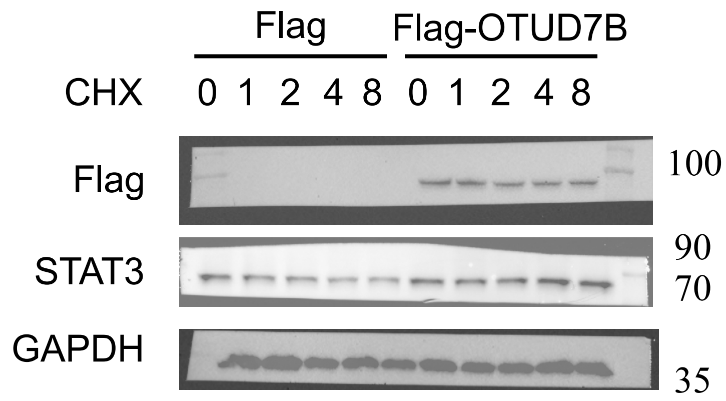

Figure S7H

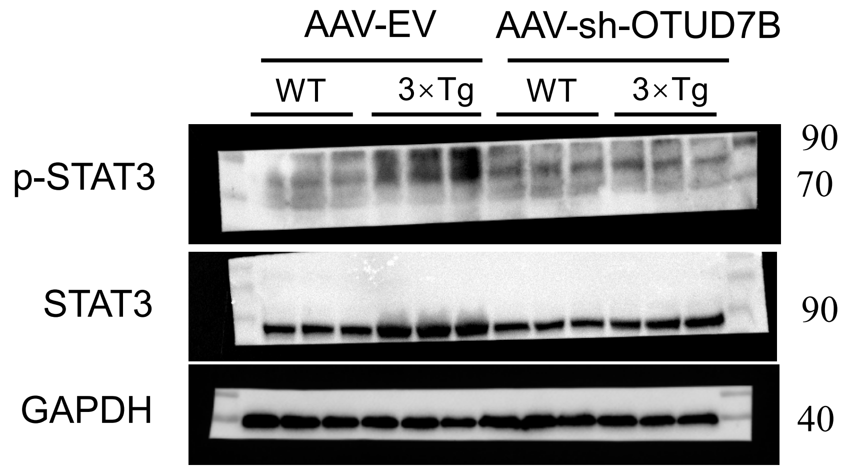

Figure S8A

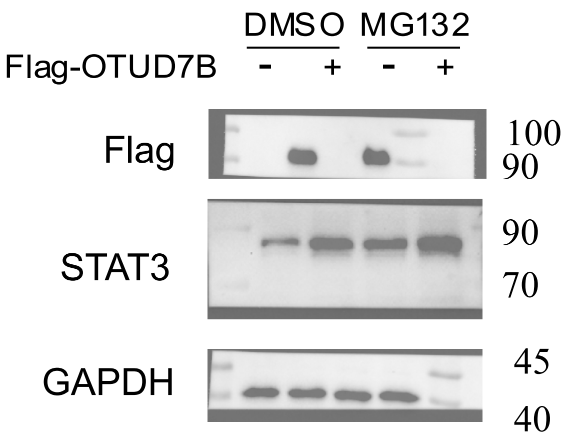

Figure S8C

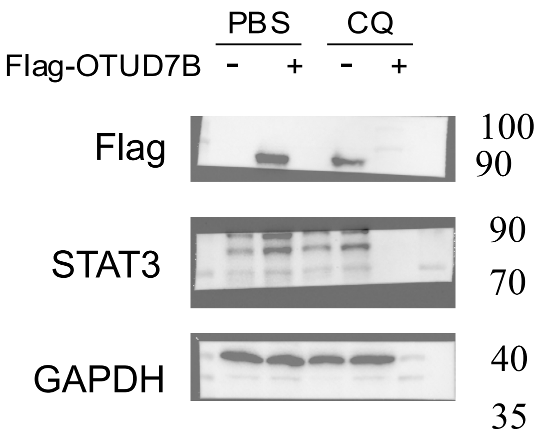

Figure 6H

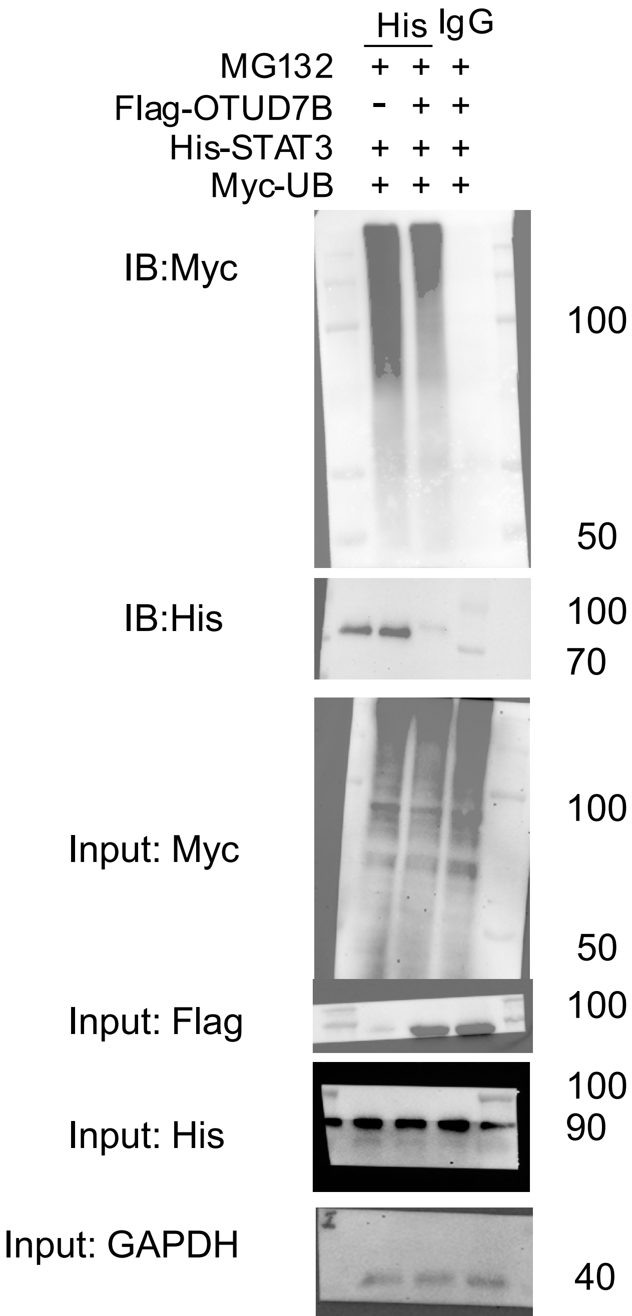

Figure 6I

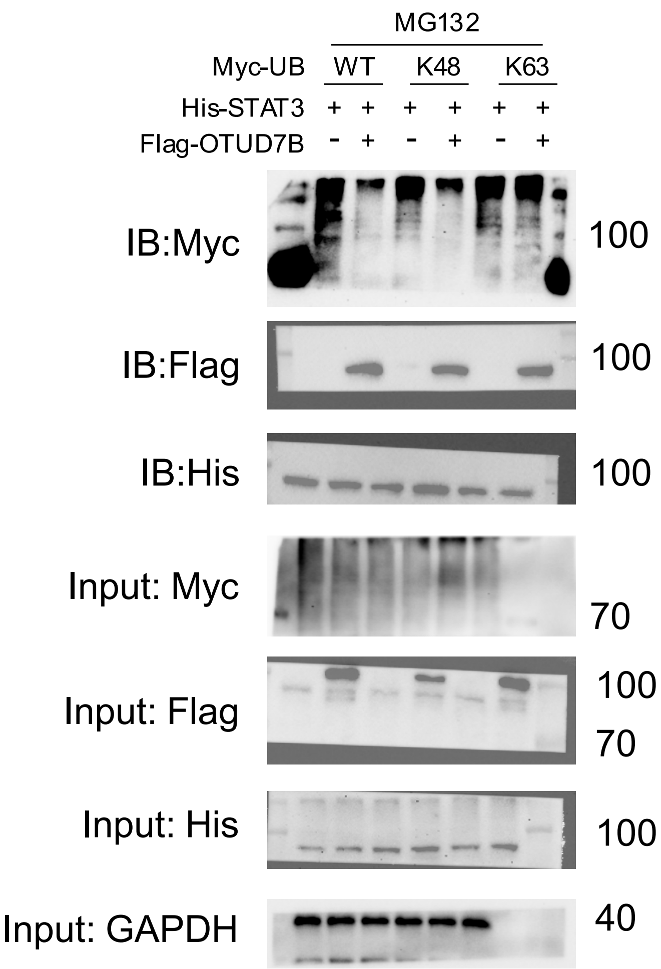

Figure 6J

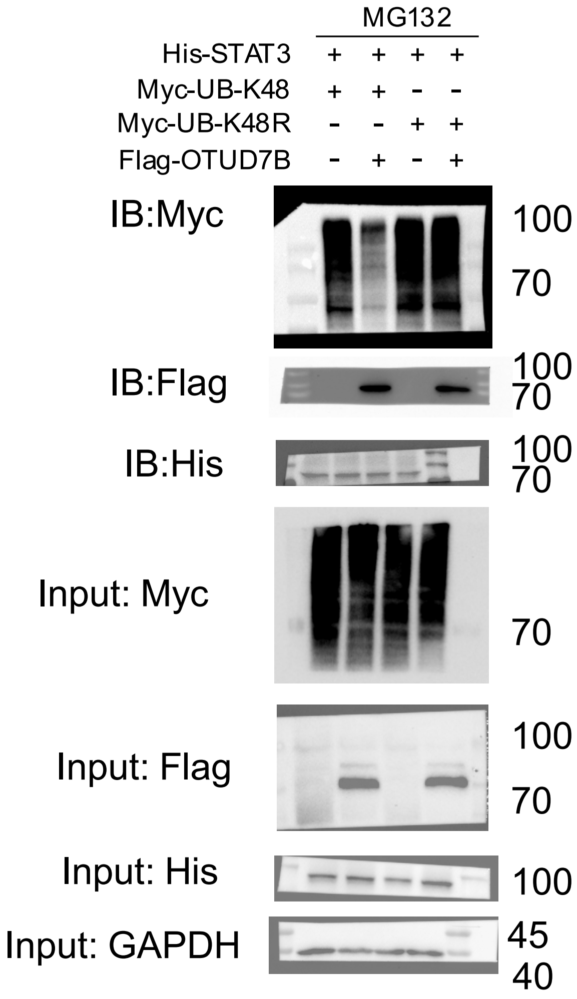

Figure 6L

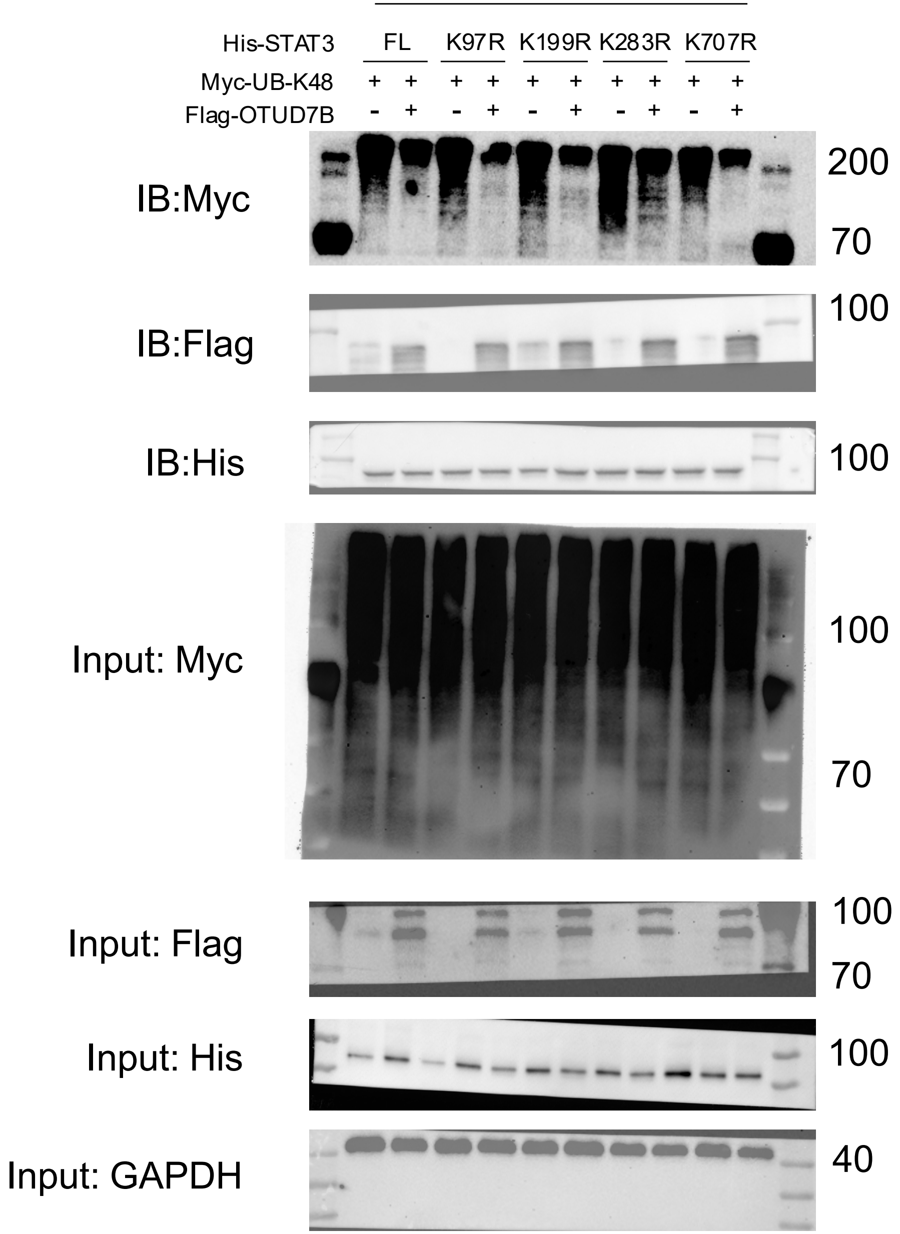

Figure S9A

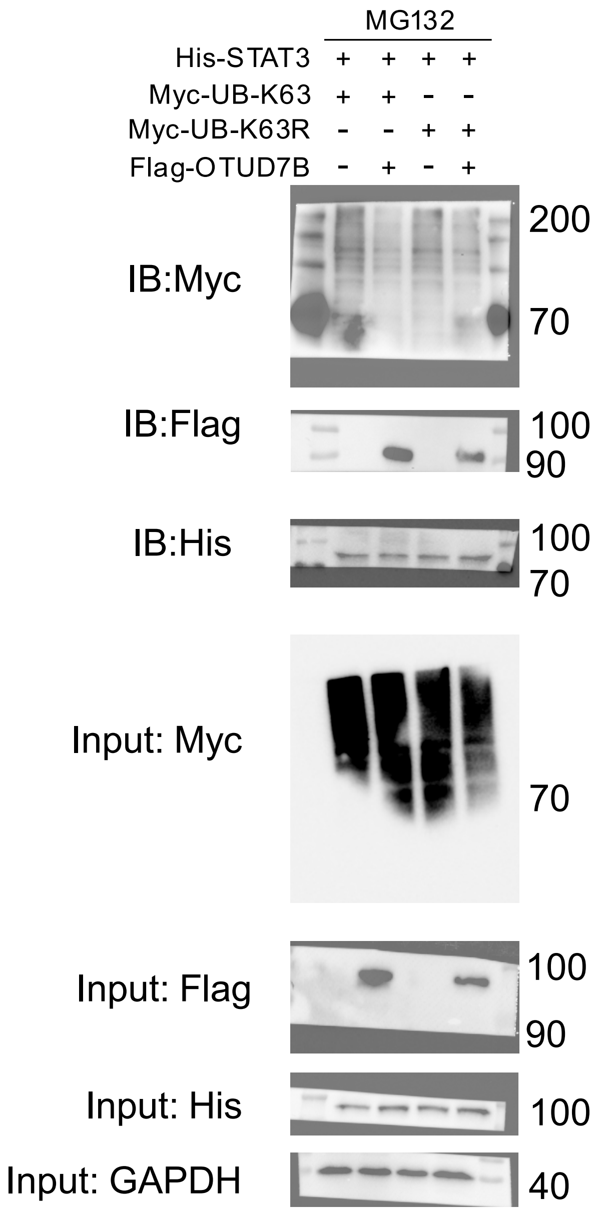

Figure S9B

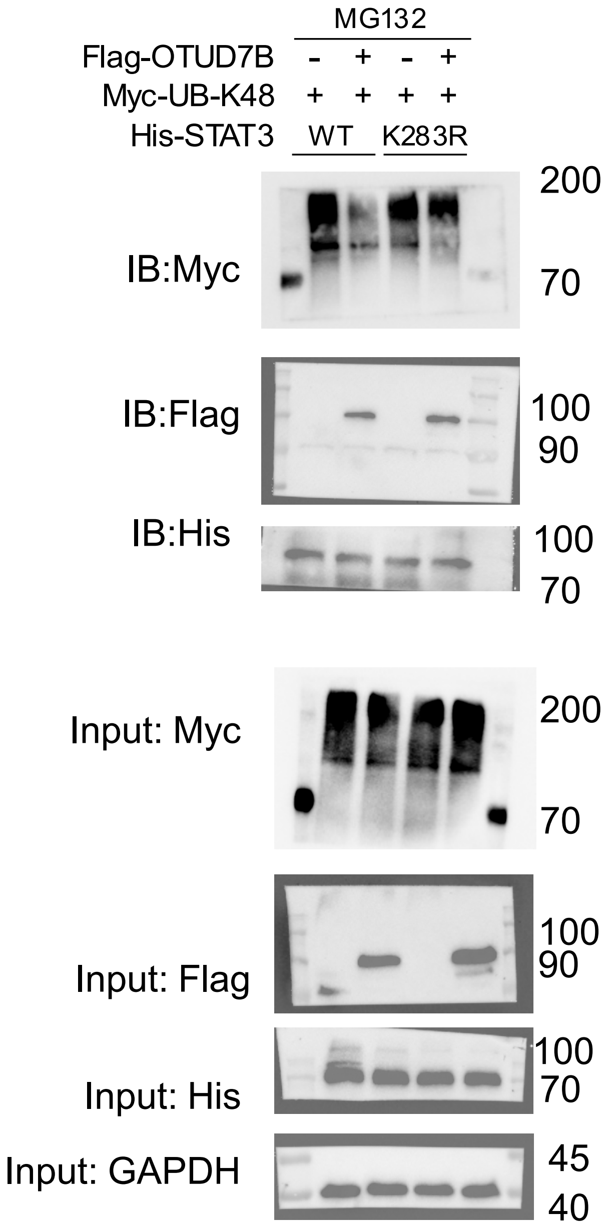

Figure 7A

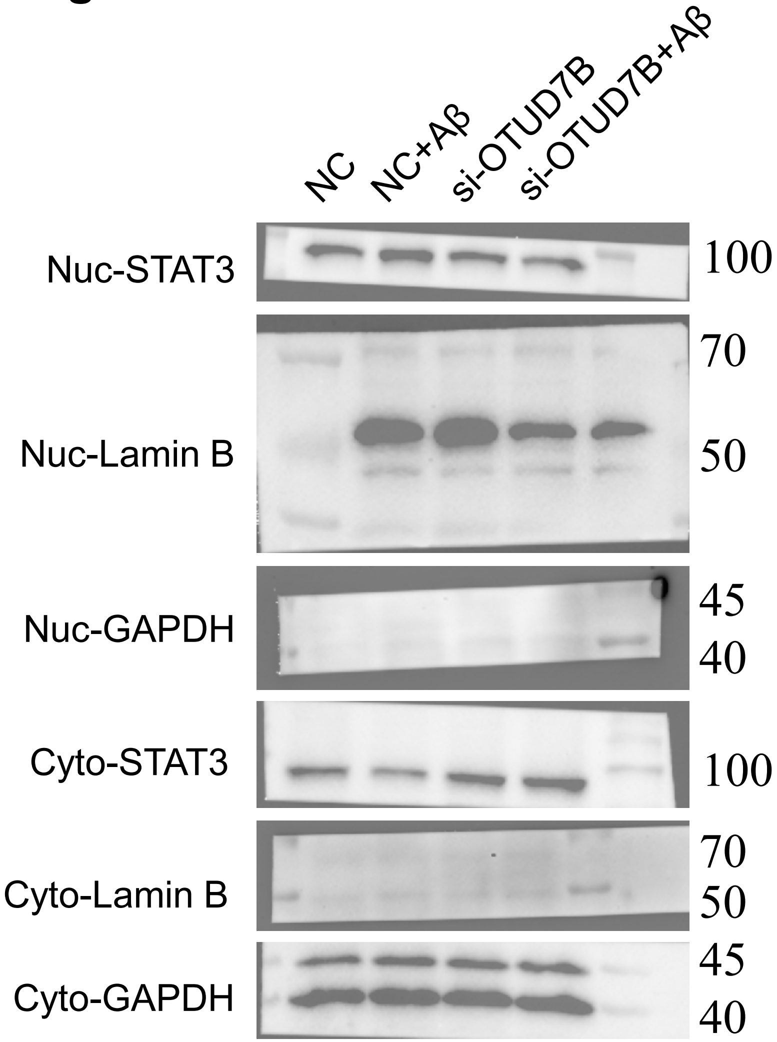

Figure 7C

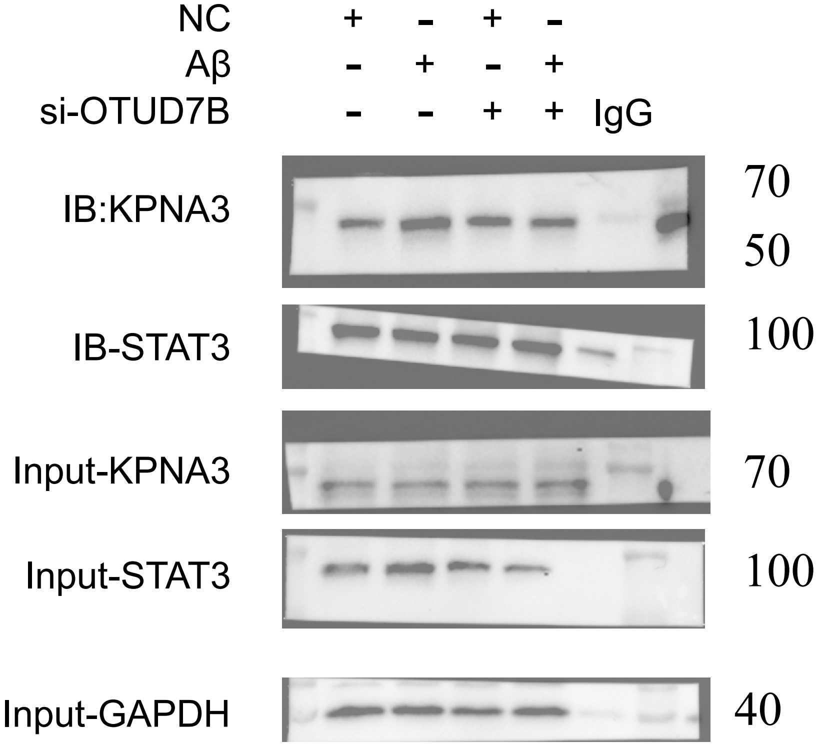

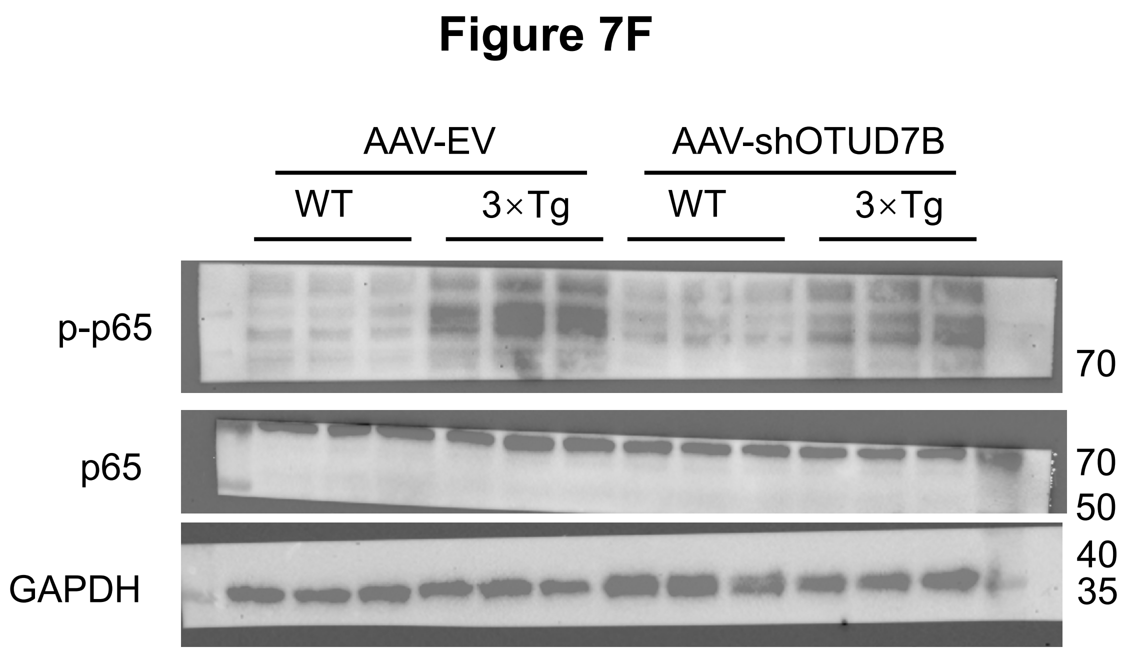

Figure 7F
AAV-EV
AAV-shOTUD7B
WT
3×Tg
WT
3×Tg
p-p65
p65
GAPDH
70
70
50
40
35

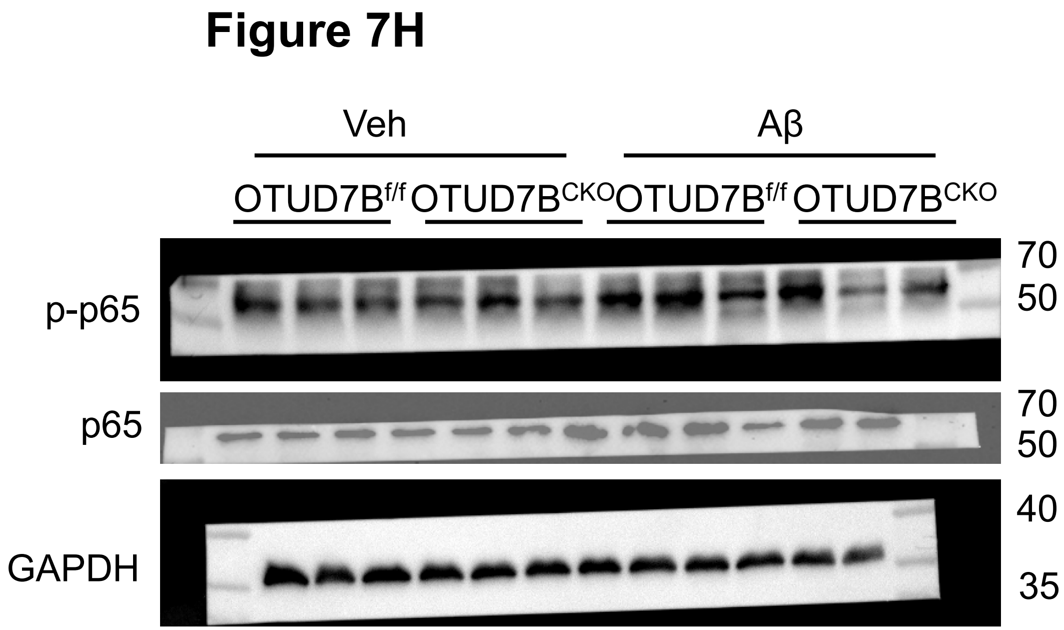

Figure 7H
Veh
Aβ
OTUD7B f/f
OTUD7B CKO
OTUD7B f/f
OTUD7B CKO
p-p65
p65
GAPDH
70
50
70
50
40
35

Figure 8C

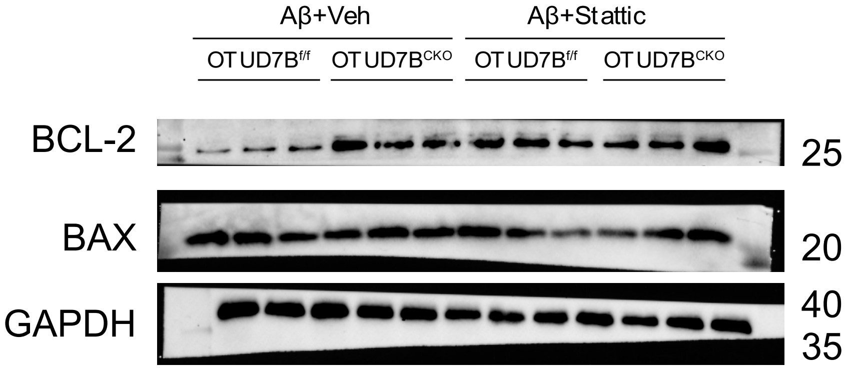

Figure S10A

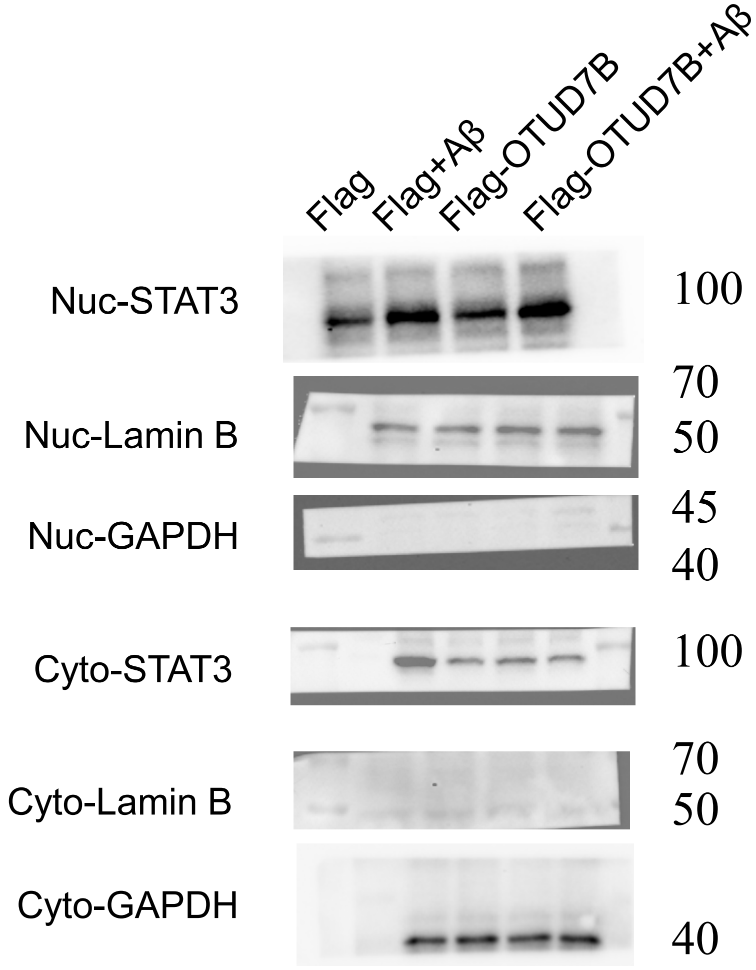

Figure S10C

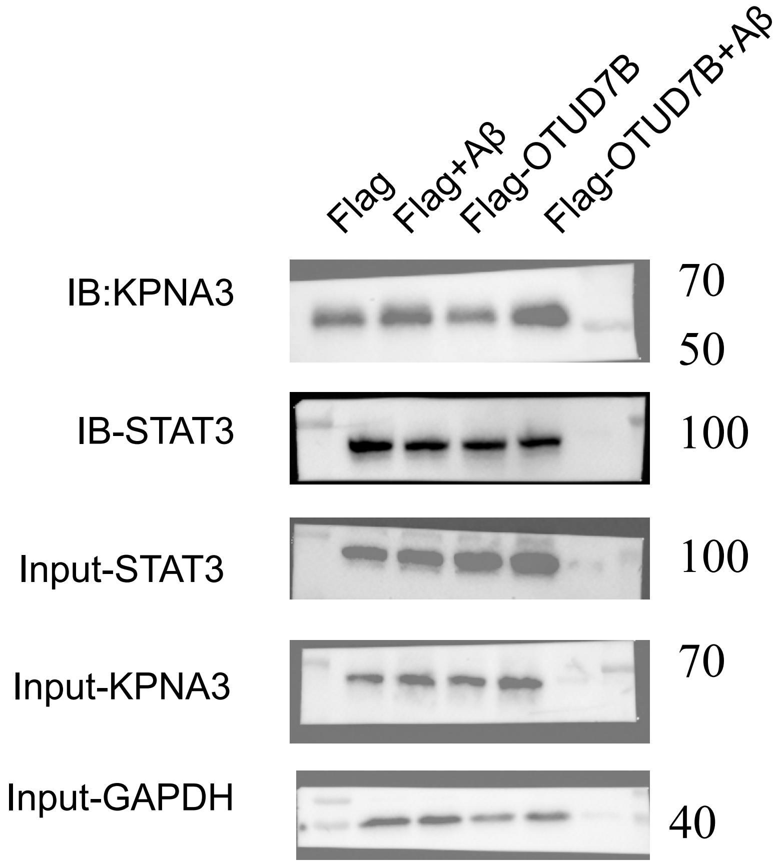

Supplement: Supplementary file 2 [file ADVS-9999-e23043-s001.zip › WB raw data_20260710.pdf]
